# Obesity, clinical, and genetic predictors for glycemic progression in Chinese patients with type 2 diabetes: A cohort study using the Hong Kong Diabetes Register and Hong Kong Diabetes Biobank

**DOI:** 10.1371/journal.pmed.1003209

**Published:** 2020-07-28

**Authors:** Guozhi Jiang, Andrea O. Luk, Claudia H. T. Tam, Eric S. Lau, Risa Ozaki, Elaine Y. K. Chow, Alice P. S. Kong, Cadmon K. P. Lim, Ka Fai Lee, Shing Chung Siu, Grace Hui, Chiu Chi Tsang, Kam Piu Lau, Jenny Y. Y. Leung, Man-wo Tsang, Grace Kam, Ip Tim Lau, June K. Li, Vincent T. Yeung, Emmy Lau, Stanley Lo, Samuel K. S. Fung, Yuk Lun Cheng, Chun Chung Chow, Ewan R. Pearson, Wing Yee So, Juliana C. N. Chan, Ronald C. W. Ma, Hong Kong Diabetes Register TRS Study Group, Hong Kong Diabetes Biobank Study Group

**Affiliations:** 1 Department of Medicine and Therapeutics, The Chinese University of Hong Kong, Prince of Wales Hospital, Hong Kong, China; 2 Hong Kong Institute of Diabetes and Obesity, The Chinese University of Hong Kong, Hong Kong, China; 3 Li Ka Shing Institute of Health Sciences, The Chinese University of Hong Kong, Hong Kong, China; 4 CUHK-SJTU Joint Research Centre in Diabetes Genomics and Precision Medicine, The Chinese University of Hong Kong, Hong Kong, China; 5 Asia Diabetes Foundation, Hong Kong, China; 6 Department of Medicine and Geriatrics, Kwong Wah Hospital, Hong Kong, China; 7 Diabetes Centre, Tung Wah Eastern Hospital, Hong Kong, China; 8 Diabetes and Education Centre, Alice Ho Miu Ling Nethersole Hospital, Hong Kong, China; 9 North District Hospital, Hong Kong, China; 10 Department of Medicine and Geriatrics, Ruttonjee Hospital, Hong Kong, China; 11 Department of Medicine and Geriatrics, United Christian Hospital, Hong Kong, China; 12 Tseung Kwan O Hospital, Hong Kong, China; 13 Department of Medicine, Yan Chai Hospital, Hong Kong, China; 14 Centre for Diabetes Education and Management, Our Lady of Maryknoll Hospital, Hong Kong, China; 15 Department of Medicine, Pamela Youde Nethersole Eastern Hospital, Hong Kong, China; 16 Department of Medicine and Geriatrics, Princess Margaret Hospital, Hong Kong, China; 17 Department of Medicine, Alice Ho Miu Ling Nethersole Hospital, Hong Kong, China; 18 Division of Population Health and Genomics, School of Medicine, University of Dundee, Dundee, Scotland, United Kingdom; University of Cambridge, UNITED KINGDOM

## Abstract

**Background:**

Type 2 diabetes (T2D) is a progressive disease whereby there is often deterioration in glucose control despite escalation in treatment. There is significant heterogeneity to this progression of glycemia after onset of diabetes, yet the factors that influence glycemic progression are not well understood. Given the tremendous burden of diabetes in the Chinese population, and limited knowledge on factors that influence glycemia, we aim to identify the clinical and genetic predictors for glycemic progression in Chinese patients with T2D.

**Methods and findings:**

In 1995–2007, 7,091 insulin-naïve Chinese patients (mean age 56.8 ± 13.3 [SD] years; mean age of T2D onset 51.1 ± 12.7 years; 47% men; 28.4% current or ex-smokers; median duration of diabetes 4 [IQR: 1–9] years; mean HbA1c 7.4% ± 1.7%; mean body mass index [BMI] 25.3 ± 4.0 kg/m^2^) were followed prospectively in the Hong Kong Diabetes Register. We examined associations of BMI and other clinical and genetic factors with glycemic progression defined as requirement of continuous insulin treatment, or 2 consecutive HbA1c ≥8.5% while on ≥2 oral glucose-lowering drugs (OGLDs), with validation in another multicenter cohort of Hong Kong Diabetes Biobank. During a median follow-up period of 8.8 (IQR: 4.8–13.3) years, incidence of glycemic progression was 48.0 (95% confidence interval [CI] 46.3–49.8) per 1,000 person-years with 2,519 patients started on insulin. Among the latter, 33.2% had a lag period of 1.3 years before insulin was initiated. Risk of progression was associated with extremes of BMI and high HbA1c. On multivariate Cox analysis, early age at diagnosis, microvascular complications, high triglyceride levels, and tobacco use were additional independent predictors for glycemic progression. A polygenic risk score (PRS) including 123 known risk variants for T2D also predicted rapid progression to insulin therapy (hazard ratio [HR]: 1.07 [95% CI 1.03–1.12] per SD; *P* = 0.001), with validation in the replication cohort (HR: 1.24 [95% CI 1.06–1.46] per SD; *P* = 0.008). A PRS using 63 BMI-related variants predicted BMI (beta [SE] = 0.312 [0.057] per SD; *P* = 5.84 × 10^−8^) but not glycemic progression (HR: 1.01 [95% CI 0.96–1.05] per SD; *P* = 0.747). Limitations of this study include potential misdiagnosis of T2D and lack of detailed data of drug use during follow-up in the replication cohort.

**Conclusions:**

Our results show that approximately 5% of patients with T2D failed OGLDs annually in this clinic-based cohort. The independent associations of modifiable and genetic risk factors allow more precise identification of high-risk patients for early intensive control of multiple risk factors to prevent glycemic progression.

## Author summary

**Why was this study done?:**

- There is marked heterogeneity in the rate of progression to insulin requirement in patients with type 2 diabetes (T2D). Whereas some patients can maintain optimal glycemic control with oral glucose-lowering drugs for prolonged periods, others experience rapid deterioration in glycemia, requiring early insulin treatment.
- It is important to gain insight into what factors are associated with glycemic progression. Previous studies identified baseline HbA1c, young age, and weight gain as independent clinical predictors for glycemic progression in White populations, but there is a paucity of data in Asians.
- Besides environmental and lifestyle factors including obesity, it is well known that genetic factors play a pivotal role in the onset of diabetes. However, it remains unclear whether genetic variants of body mass index (BMI) and T2D predicts glycemic progression once T2D develops. Moreover, genetic variants associated with response to oral glucose-lowering drugs might also predict glycemic progression.

**What did the researchers do and find?:**

- We investigated glycemic progression among 7,091 Chinese patients with T2D from the Hong Kong Diabetes Register enrolled between 1995 and 2007. We explored the associations of clinical variables and different polygenic risk scores (PRSs) of obesity, T2D, and response to oral glucose-lowering drugs with glycemic progression and validated the associations of PRSs in another multicenter prospective cohort of Hong Kong Diabetes Biobank (HKDB).
- We found that there was an approximately 1.3-year delay of insulin use after oral glucose-lowering drug failure in real-world practice.
- We observed a U-shape association between BMI and glycemic progression and found that young age of diagnosis, high HbA1c and triglyceride, use of tobacco, and presence of microvascular complications also predicted rapid glycemic progression.
- We found that the PRS derived from 123 known risk variants for T2D was associated with early age of diagnosis and rapid glycemic progression, with validation in the replication cohort of HKDB, whereas the PRS derived from 63 BMI-related variants was associated with BMI but not glycemic progression.

**What do these findings mean?:**

- This study highlighted the delay in insulin treatment after oral glucose-lowering drug failure, which represents an important treatment gap in clinical practice.
- This work emphasized the importance of both genetic and modifiable risk factors, notably lipo-glucotoxicity, obesity, and tobacco use on glycemic progression, which enables identification of high-risk patients for optimal control of risk factors to improve glycemic durability.
- This study provides novel insights toward the underlying pathophysiology underlining glycemic progression, highlighting some overlap with pathogenesis of T2D.
- Our work highlights the potential utility of incorporating PRSs for drug response to guide clinical management of T2D.

## Introduction

Type 2 diabetes (T2D) is a progressive and multisystemic disease with hyperglycemia as a major risk factor for complications [1]. Delayed intervention, patient nonadherence, heterogeneity of phenotypes and treatment responses can lead to different trajectories in treatment escalation. Whereas some patients can be controlled with oral glucose-lowering drugs (OGLDs) for decades, others experience rapid deterioration in glycemia, requiring early insulin treatment [2]. In Whites with T2D, baseline HbA1c, young age, and weight gain independently predicted glycemic progression [3]. In the IMI-DIRECT study, researchers used time to insulin initiation as an index of glycemic progression and reported that patients with low body mass index (BMI) <24 kg/m^2^ and BMI >30 kg/m^2^ were susceptible to rapid glycemic progression [2].

Genetic factors play a pivotal role in the onset of diabetes although this is strongly influenced by environmental and lifestyle factors, notably obesity. The heritability of T2D ranges from 30% to 70% in different populations with the discovery of a large number of genetic variants mainly implicated in beta-cell biology [4]. Although genetic analysis also supports the causal role of obesity in T2D [5], it is unclear whether genetic variants of BMI and T2D predict glycemic progression once T2D develops. In the IMI-DIRECT study, a high polygenic risk score (PRS) comprising 61 risk variants of T2D was associated with a young age at diagnosis but not time to insulin requirement in European patients [2]. Furthermore, pharmacogenetic studies have identified variants associated with poor response to OGLD, such as metformin [6–13], sulphonylurea (SU) [14–18], and thiazolidinediones (TZD) [19–21].

We hypothesize that clinical and genetic variants associated with T2D and response to OGLDs predict glycemic progression in Asian patients with T2D. These predictors may identify high-risk patients for early intensification and individualization of treatment to prevent glycemic progression and development of complications. In a prospective cohort with documentation of clinical and genetic profiles and treatment progression, we explored the associations of clinical variables and different PRSs of obesity, T2D, and response to OGLDs with glycemic progression in insulin-naïve Chinese patients with T2D.

## Materials and methods

### Study population

This study utilized data from the Hong Kong Diabetes Register (HKDR) established in 1995 at the Prince of Wales Hospital (PWH), the teaching hospital of the Chinese University of Hong Kong [22,23]. The HKDR consecutively enrolled patients referred to the Diabetes Mellitus and Endocrine Centre for comprehensive assessment of complications and metabolic control. Referral sources included hospital-based specialty clinics, community clinics, and general practitioners. Once registered, patients were observed to the time of death. Ethical approval was obtained from the Clinical Research Ethics Committees of the Chinese University of Hong Kong. Written informed consent was obtained from all participants at the time of enrollment for collection of clinical information and biosamples for archival and research purposes. The recruitment methods, definitions, and biochemical investigations have been described [22,24].

Between 1995 and December 31, 2007, a consecutive cohort consisting of 10,129 patients with diabetes was assessed. After sequentially excluding 61 patients with non-Chinese or unknown ethnicity, 400 with type 1 diabetes (T1D) or missing data on type of diabetes, 2,544 with insulin use or considered having OGLD failure at baseline (see definition below), and 33 requiring insulin within 1 year, we prospectively curated a primary cohort of 7,091 Chinese patients with detailed information on risk factors, complications, drug use, and clinical outcomes for further analysis (S1 Fig).

For validation of genetic association, we used an independent prospective cohort from Hong Kong Diabetes Biobank (HKDB), which recruited patients from 11 diabetes centers at major public hospitals across Hong Kong since 2014. Recruitment and assessment methods were similar to that in HKDR. A total of 6,914 unrelated patients from HKDB were genotyped. In this replication cohort, after excluding 162 patients with non-Chinese or non-T2D, 2,675 with insulin use at baseline, 3 requiring insulin within 1 year, and 135 patients who overlapped with HKDR, we included 3,939 patients with T2D for this replication study (S2 Fig). All patients gave written informed consent for DNA collection and data analysis for research purposes. Genetic data from HKDB became available for replication analysis around April 2019.

### Baseline clinical and laboratory measurements

Clinical assessment was performed based on a modified European DiabCare protocol [25]. Details of assessment methods and definitions of clinical outcomes have been described [24,26]. In brief, all patients in the HKDR and HKDB underwent clinical assessments and laboratory investigations after 8-hour overnight fast, including eye, feet, urine, and blood examinations. Eye examination included visual acuity and fundoscopy through dilated pupils or retinal photography. Retinopathy was defined by typical changes due to diabetes, laser scars, or a history of vitrectomy. Foot examination was performed using Doppler ultrasound scan and monofilament and graduated tuning fork. Fasting blood was sampled for measurement of plasma glucose, HbA1c, and lipid profile (total cholesterol, high-density lipoprotein cholesterol [HDL-C], triglycerides, and calculated low-density lipoprotein cholesterol [LDL-C]), and random spot urinary sample was used to assess albumin-to-creatinine ratio (ACR). The Chronic Kidney Disease Epidemiology Collaboration equation was used to estimate glomerular filtration rate (eGFR) [27].

### Definition of clinical outcomes

Clinical outcomes were defined using hospital discharge diagnoses based on the *International Classification of Diseases*, *Ninth Revision* (ICD-9) and mortality as censored on or before June 30, 2014. The Hong Kong Hospital Authority Central Computer System records admissions to all public hospitals, which provides about 95% of inpatient bed-days in Hong Kong. All hospitalization records were retrieved from this system using a unique identifier number. Given the frequent delay in starting insulin due to patients’ reluctance or clinicians’ delayed decision, we used a composite end point for “requirement of insulin treatment” to define glycemic progression, (1) progression to continuous insulin treatment (more than 6 months’ duration), or (2) failure of OGLDs (2 consecutive HbA1c ≥8.5%, more than 3 months apart during treatment with ≥2 OGLDs [metformin, SU, or TZD]), in line with the definition used in the IMI-DIRECT study [2]. Follow-up time was defined as the period from baseline visit to the date of the first clinical end point or the censored dates, whichever came first. Since the OGLD data during follow-up in the HKDB were not available for this analysis, we used continuing insulin treatment as the end point in the replication cohort.

### PRS

In a subset of 4,423 patients from the primary cohort with genome-wide association study (GWAS) data, we developed 6 different PRSs using published genetic variants associated with T2D, BMI, or response to OGLDs. We investigated the associations between glycemic progression and each PRS, including (1) European-T2D PRS derived from 143 single nucleotide polymorphisms (SNPs) identified in a meta-analysis of GWAS in European populations [4], (2) Asian-T2D PRS derived from 48 SNPs including those identified in Asian-GWAS or European SNPs replicated in East Asians [28], (3) BMI PRS derived from 97 SNPs associated with BMI [29,30], (4) metformin PRS derived from 8 SNPs associated with metformin response [6–13], (5) SU PRS derived from 7 SNPs associated with SU response [14–18], and (6) TZD PRS derived from 3 SNPs associated with TZD response [19–21]. All SNPs in PRS associated with BMI and drug responses were discovered in European populations.

We extracted these SNPs from imputed genotyping data using the Illumina Omni 2.5+ exome array (Illumina, San Diego, CA). We selected independent common SNPs (linkage disequilibrium [LD] coefficient r^2^ < 0.5; minor allele frequency [MAF] > 0.01) available in our dataset with good genotyping quality to construct the final PRSs. Standard quality control (MAF > 0.01; call rate > 97%; *P* > 0.0001 in Hardy-Weinberg equilibrium) was performed and genotype data were imputed using minimac3 with the 1000 Genomes Project phase 3 v5 as reference panel [23]. Finally, we obtained 123, 48, 63, 8, 7, and 3 independent common SNPs to develop the European-T2D, Asian-T2D, BMI, metformin, SU, and TZD PRSs, respectively (S1–S6 Tables). Because of the small number of SNPs associated with each OGLD, we created a combined PRS using all 18 SNPs for drug responses. Each PRS was first constructed by summing the score of reported risk allele for each SNP based on an additive genetic model and then rescaled to a score to express the standard deviation (SD) using the following formula: (individual PRS value − population mean PRS) ÷ population SD of PRS. The final analysis is based on (1) the genetic risk per SD of the standardized PRS and (2) tertile analysis of the PRS for glycemic progression (tertile 1: low; tertile 2: intermediate, and tertile 3: high).

In the replication cohort, we performed genotyping using the Illumina Infinium Global Screening Array (GSA) and conducted quality control and imputation using similar criteria. A proxy SNP with r^2^ >0.6 (according to the 1000 Genome CHB panel) was selected when the index SNP was imputed with poor quality. One European-T2D variant (rs17405722 near *STAT3* gene) with poor proxies was dropped, and 1 SU variant (rs1801278 in *IRS1* gene) was replaced with a proxy variant (rs56171406; r^2^ = 1).

### Statistical analysis

We used Cox proportional hazards regression model for prospective analysis. We examined the Cox proportional hazards assumption by plotting the scaled Schoenfeld residuals against log-transformed time for each covariate and further tested the assumption via the test proposed by Grambsch and Therneau [31]. As suggested by reviewers, we used 3-knot restricted cubic spline to plot the relationship between BMI as a continuous variable (rather than BMI categories) and glycemic progression. Because of the nonlinear relationship between BMI and rate of progression to insulin requirement, we divided BMI into 4 groups using the World Health Organization (WHO) Asian definition for obesity—underweight (BMI < 18.5 kg/m^2^), normal (≥18.5–23.0 kg/m^2^), overweight (≥23.0–25.0 kg/m^2^), and obese (≥25.0 kg/m^2^)—for the survival analysis [32]. We assessed crude survival (for non-insulin requirement) with the Kaplan-Meier estimator, stratified for BMI categories. Since the proportional hazard assumption for baseline HbA1c was not met, we stratified the cohort into 3 groups (HbA1c < 7%, ≥ 7–9%, and ≥ 9%) to allow for a different hazard function in each group. Both BMI and HbA1c categories were included as strata variables in Cox models, whereas other covariates were considered to have same effect across strata. A procedure of stepwise model selection by the Akaike information criterion (AIC) was employed to identify clinical predictors for glycemic progression in multivariate analysis. Linear regression was used to examine associations between PRSs and quantitative traits.

All data were expressed as percentages; means and SDs; or medians and interquartile ranges (IQRs) as appropriate. All statistical tests were 2-sided, and a *P* value <0.05 was considered to be significant. Analyses were performed using R (version 3.1.0; http://www.R-project.org).

## Results

### Baseline characteristics of the study population

Among 7,091 patients included in the analysis (mean age 56.8 ± 13.3[SD] years; 47% men; median duration of diabetes 4 [IQR: 1–9] years), 3,111 (43.9%) had glycemic progression during a median follow-up period of 8.8 (IQR: 4.8–13.3) years. The incident rate of glycemic progression was 48.0 (95% confidence interval [CI] 46.3–49.8) per 1,000 person-years. The median period from diagnosis of T2D to glycemic progression was 13.7 (IQR: 9.3–18.7) years. Among the progressors, 2,519 were actually started on insulin, with 33.2% of them (*n* = 836) experiencing a median lag period of 1.3 (IQR: 0.4–3.0) years before insulin was initiated. Compared with nonprogressors, patients with glycemic progression had younger age of diagnosis; longer duration of diabetes; and higher BMI, HbA1c, triglyceride, LDL-C, and urinary ACR and were more likely to be smokers and have microvascular complications (Table 1). In the replication cohort comprising 3,939 patients, 172 (4.4%) had glycemic progression during a median follow-up period of 1.9 (IQR: 1.5–2.3) years. Progressors had younger age of diagnosis, longer duration of diabetes, and higher HbA1c and triglyceride (S7 Table).

**Table 1:** Baseline characteristics of progressors and nonprogressors for glycemic deterioraton, defined as need for insulin treatment in the primary cohort.

Characteristics	Nonprogressors	Progressors	*P*
*N*	3,980	3,111	
Age (year)	58.28 ± 13.09	54.84 ± 13.23	<0.001
Age at diagnosis (year)	53.4 ± 12.52	48.18 ± 12.35	<0.001
Young age at diagnosis (<40 years)	14.35% (558)	25.28% (778)	<0.001
Year of diagnosis	1996 (1992–2002)	1994 (1989–1998)	<0.001
Male sex	45.95% (1,829)	47.19% (1,468)	0.302
Duration of diabetes (year)	3 (1–8)	5 (2–10)	<0.001
Smoking status			<0.001
Former	15.38% (609)	16.18% (502)	
Current	10.98% (435)	14.89% (462)	
BMI (kg/m^2^)	25.18 ± 3.92	25.39 ± 4.11	0.031
HbA1c (%)	6.87 ± 1.39	8.02 ± 1.86	<0.001
Triglyceride (mmol/L)	1.34 (0.95–1.96)	1.47 (1.01–2.2)	<0.001
HDL-C (mmol/L)	1.34 ± 0.36	1.28 ± 0.35	<0.001
LDL-C (mmol/L)	3.05 ± 0.95	3.21 ± 1	<0.001
Systolic BP (mmHg)	134.17 ± 20.24	134.72 ± 20.63	0.265
Diastolic BP (mmHg)	75.6 ± 11.07	76.71 ± 10.81	<0.001
Urinary ACR (mg/mmol)	1.37 (0.64–4.98)	2.26 (0.86–10.9)	<0.001
eGFR (mL min^−1^ per 1.73 m^2^)	104.84 (86.51–123.78)	108.04 (87–130.26)	<0.001
Sensory neuropathy	16.38% (652)	22.18% (690)	<0.001
Retinopathy	17.11% (681)	25.2% (784)	<0.001
Stroke history	3.02% (120)	1.51% (47)	<0.001
CKD history	10.1% (402)	11.12% (346)	0.165
PVD history	4.75% (189)	4.98% (155)	0.65
CHD history	7.09% (282)	6.27% (195)	0.173
Baseline drug treatment			
Lipid-lowering drugs	17.19% (684)	13.69% (426)	<0.001
BP-lowering drugs	44.1% (1,755)	38.67% (1,203)	<0.001
ACEIs or ARBs	17.56% (699)	17.9% (557)	0.709
Oral glucose-lowering drugs	62.74% (2,497)	70.43% (2,191)	<0.001

Data are expressed as mean ± standard deviation, percentage (number), or median (interquartile range); *t* test or Mann-Whitney rank sum test was used for the continuous variables, and χ^2^ test was used for the categorical variables.

Abbreviations: ACEI, angiotensin-converting enzyme inhibitor; ACR, albumin-to-creatinine ratio; ARB, angiotensin receptor block; BMI, body mass index; BP, blood pressure; CHD, coronary heart disease; CKD, chronic kidney disease; eGFR, estimated glomerular filtration rate; HDL-C, high-density lipoprotein cholesterol; LDL-C, low-density lipoprotein cholesterol; PVD, peripheral vascular disease

### Association of clinical factors with diabetes progression

**Fig 1:**
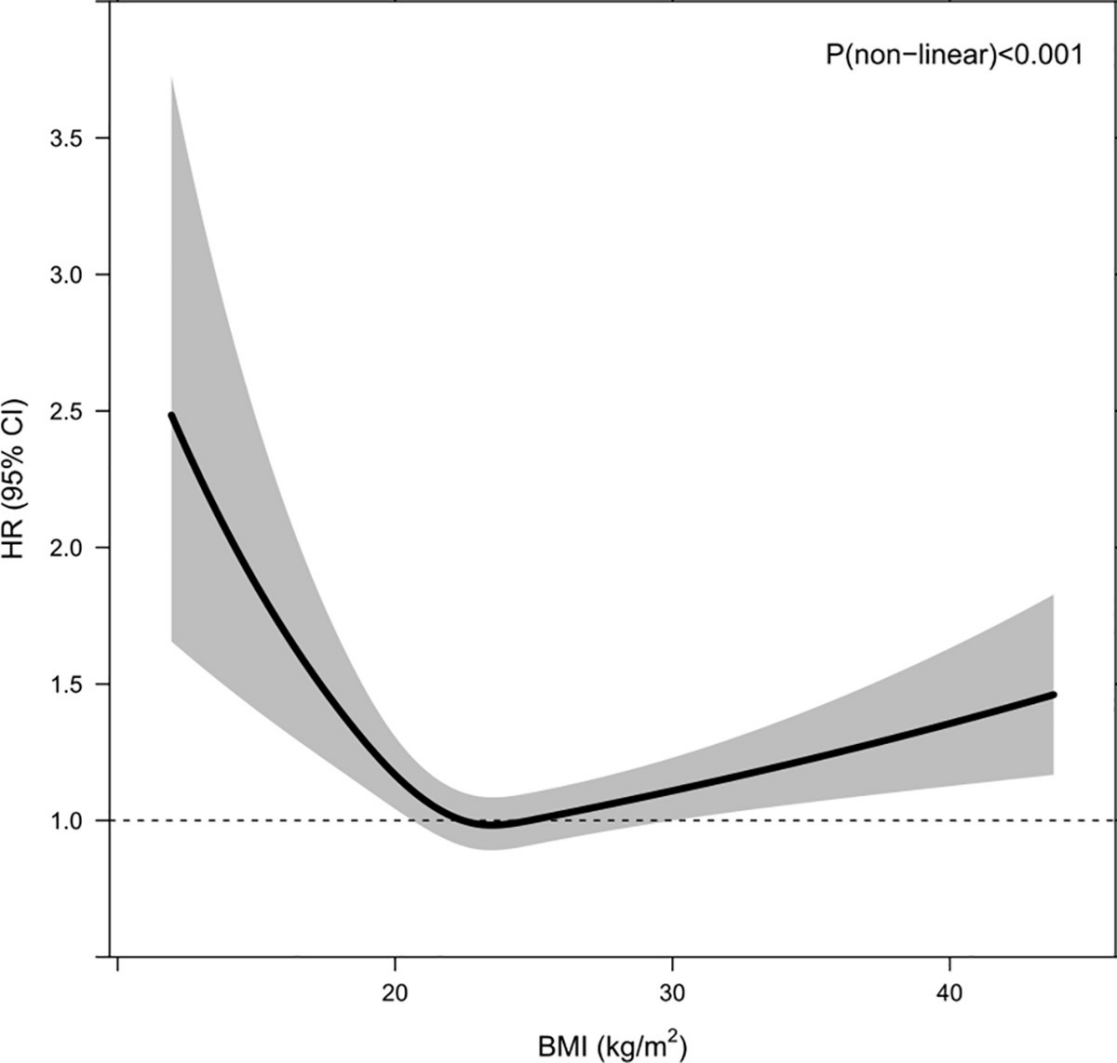
HRs of BMI as a continuous variable and glycemic progression. The dashed line is the reference line at HR = 1. The solid curve and the shaded areas stand for the HRs and their 95% CIs for glycemic progression, respectively. HRs were adjusted for age at diagnosis, gender, duration of diabetes, year of diagnosis, smoking status, LDL-C, HbA1c, log triglyceride, log urinary ACR, eGFR, retinopathy, sensory neuropathy, history of chronic kidney disease, and use of different medications (yes/no). ACR, albumin-to-creatinine ratio; BMI, body mass index; CI, confidence interval; eGFR, estimated glomerular filtration rate; HR, hazard ratio; LDL-C, low-density lipoprotein cholesterol.

**Fig 2:**
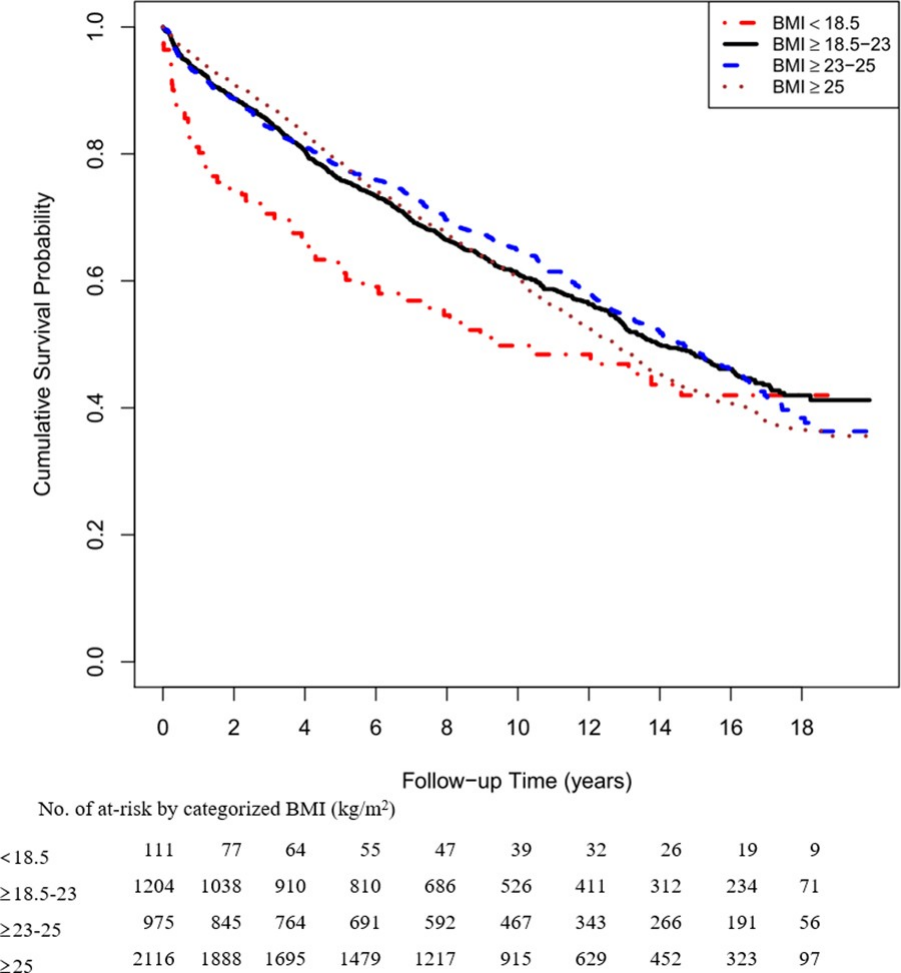
Survival curves for 4 BMI categories according to the World Health Organization definition for obesity in Asians. BMI, body mass index.

There was a U-shape association between BMI and glycemic progression (*P* < 0.001 for nonlinearity; Fig 1) after adjustment for age at diagnosis, gender, duration of diabetes, year of diagnosis, smoking status, LDL-C, HbA1c, triglyceride, urinary ACR, eGFR, retinopathy, sensory neuropathy, history of chronic kidney disease (CKD), and use of different medications (yes/no). Compared with those with a normal BMI of 18.5–23 kg/m^2^, patients with low BMI <18.5 kg/m^2^ had a hazard ratio (HR) of 1.66 (95% CI 1.31–2.10), and those with obesity (BMI ≥ 25.0 kg/m^2^) had an HR of 1.12 (95% CI 1.02–1.22) after adjustment for conventional risk factors. On Kaplan-Meier analysis, patients with low BMI (<18.5 kg/m^2^) had the fastest rate of glycemic progression (Fig 2). Compared with patients with HbA1c <7%, those with HbA1c of 7%–9% had an HR of 2.14 (95% CI 1.93–2.36), and those with HbA1c >9% had an HR of 4.07 (95% CI 3.63–4.56) for glycemic progression. Patients diagnosed before the age of 40 had an HR of 1.28 (95% CI 1.18–1.39), compared with those diagnosed after age of 40 years. Within the BMI and HbA1c strata, the multivariate Cox model derived from stepwise selection shows that young age at diagnosis; long disease duration; active/ex-smoking status; high urinary ACR and triglyceride; presence of retinopathy, neuropathy, and CKD; and use of OGLDs were independent predictors for glycemic progression (Table 2).

**Table 2:** Multivariate Cox proportional hazards model derived from stepwise model selection by the Akaike information criterion for diabetes progression.

Covariate	HR (95% CI)	*P*
Age at diagnosis (per 1 year)	0.98 (0.97–0.98)	<0.001
Year of diagnosis (per 1 year)	1.03 (1.01–1.05)	0.001
Duration of diabetes (per 1 year)	1.05 (1.03–1.07)	<0.001
Smoking		
Ex-smoker	1.38 (1.22–1.57)	<0.001
Current smoker	1.15 (1.01–1.32)	0.038
log (triglyceride)	1.11 (1.01–1.22)	0.029
LDL-C	0.96 (0.91–1.01)	0.131
log urinary ACR	1.15 (1.11–1.19)	<0.001
eGFR	0.998 (0.996–1.001)	0.064
Sensory neuropathy	1.27 (1.13–1.42)	<0.001
Retinopathy	1.28 (1.14–1.43)	<0.001
CKD history	1.67 (1.38–2.02)	<0.001
Use of lipid-lowering drugs	0.89 (0.77–1.04)	0.139
Use of ACEIs or ARBs	1.12 (0.98–1.27)	0.104
Use of oral glucose-lowering drugs	1.37 (1.22–1.53)	<0.001

BMI and baseline HbA1c categories were included as strata variables. BMI was categorized as 4 groups (<18.5, 18.5–23, 23–25, and ≥25 kg/m^2^) and baseline HbA1c was categorized as 3 groups (<7%, ≥7%–9%, and ≥9%).

Abbreviations: ACEI, angiotensin-converting enzyme inhibitor; ARB, angiotensin receptor block; BMI, body mass index; CI, confidence interval; CKD, chronic kidney disease; eGFR, estimated glomerular filtration rate; HR, hazard ratio; LDL-C, low-density lipoprotein cholesterol

### Association of PRS with glycemic progression

**Fig 3:**
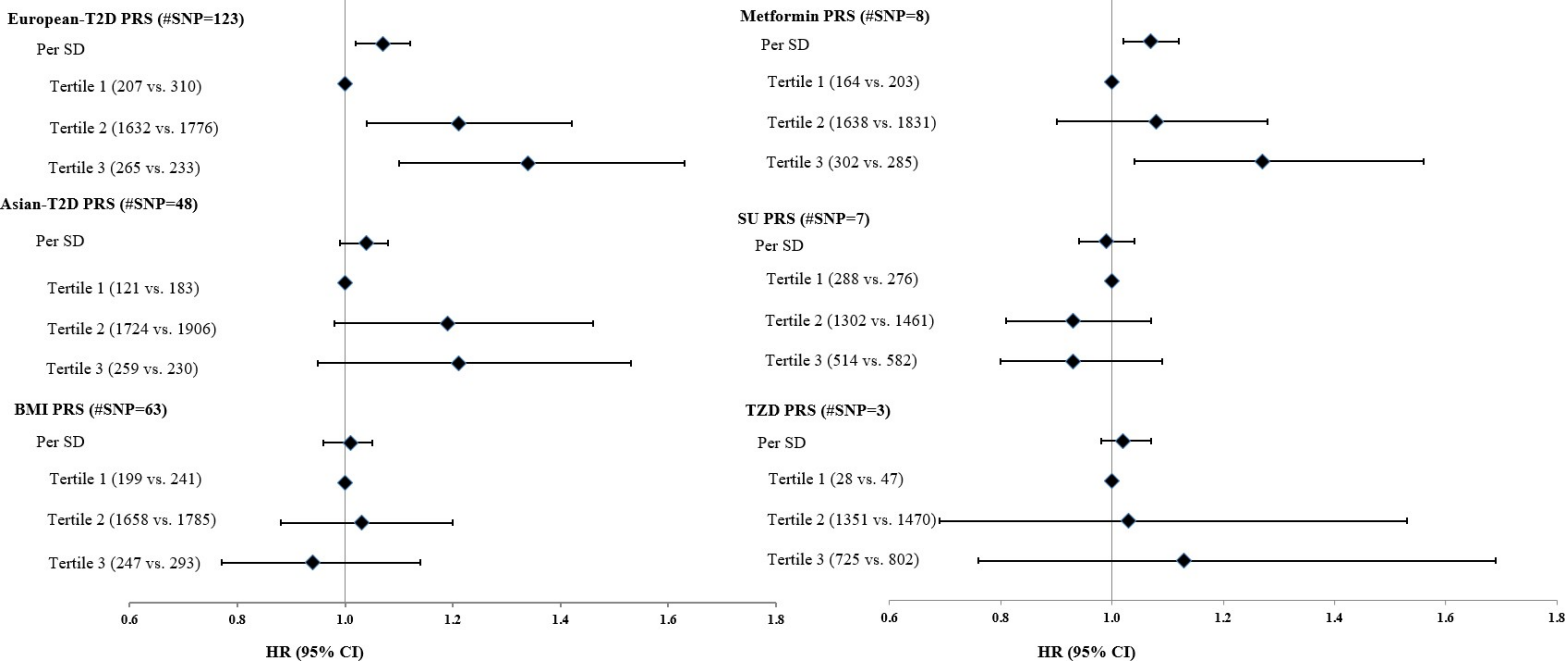
Association between PRSs and progression to requirement of insulin treatment. HRs were adjusted for all clinical risk factors identified by stepwise variable selection, including age at diagnosis, gender, duration of diabetes, year of diagnosis, smoking status, LDL-C, HbA1c, log triglyceride, log urinary ACR, eGFR, retinopathy, sensory neuropathy, history of chronic kidney disease, and use of different medications (yes/no). Number of progressors versus nonprogressors was presented in parentheses for each subgroup. ACR, albumin-to-creatinine ratio; eGFR, estimated glomerular filtration rate; HR, hazard ratio; LDL-C, low-density lipoprotein cholesterol; PRS, polygenic risk score; SD, standard deviation; SNP, single nucleotide polymorphism; SU, sulphonylurea; TZD, thiazolidinediones.

After adjusting for clinical predictors (Fig 3 and S8 Table), the PRS of 123 European-T2D SNPs was independently associated with glycemic progression (HR: 1.07 [95% CI 1.03–1.12] per SD; *P* = 0.001). Among the 3 PRSs related to drug response, the metformin PRS was associated with glycemic progression (HR: 1.07 [95% CI 1.02–1.12] per SD; *P* = 0.005). The top tertile of the composite PRS for drug response showed nominal significance with glycemic progression, compared with the reference group with a HR of 1.22 (95% CI 1.03–1.45; *P* = 0.022) (S8 Table). No significant associations were found for other PRSs or individual risk variant (S1–S6 Tables).

On multivariate linear regression with adjustment for confounding factors, the European-T2D PRS and Asian-T2D PRS were associated with earlier age at diagnosis (beta [SE] = −0.617 [0.155] per SD; *P* = 6.58 × 10^−5^ and beta [SE] = −0.817 [0.154] per SD; *P* = 1.24 × 10^−7^, respectively), whereas the BMI PRS was associated with baseline BMI (beta [SE] = 0.312 [0.057] per SD; *P* = 5.84 × 10^−8^; S8 Table).

In the replication HKDB cohort including 3,939 patients (mean age 60.9 ± 10.9 years; 59.5% men; median duration of diabetes 7 [IQR: 3–13] years), 172 (4.4%) progressed to insulin use during a median follow-up period of 1.9 (IQR: 1.5–2.3) years. The risk association of European-T2D PRS with glycemic progression was significant after adjusting for confounding factors (HR: 1.24 [95% CI 1.06–1.46] per SD; *P* = 0.008) (S9 Table) but not for the metformin PRS. The European-T2D PRS and Asian-T2D PRS were associated with age at diagnosis, whereas the BMI PRS was associated with baseline BMI (S9 Table).

### Sensitivity analysis

We performed the following sensitivity analyses to assess the robustness of our findings. First, we repeated the analysis by defining the subcomponent of glycemic progression using the end point of continuing insulin treatment. After excluding 461 non-Chinese or non-T2D patients, 2,061 insulin-treated patients at baseline, and 37 patients initiated on insulin within 1 year of enrollment, we obtained a prospective cohort of 7,570 patients for this sensitivity analysis. During a median of 9.3 (IQR: 5.4–13.6) follow-up years, 38.1% (*N* = 2,882) required continuing use of insulin. Within this cohort, 4,475 patients had genotyping data. The associations between PRSs and insulin initiation were largely unchanged. The European-T2D PRS and metformin PRS were associated with progression to insulin use (HR: 1.07 [95% CI 1.02–1.12] per SD, *P* = 0.004; 1.06 [95% CI 1.01–1.11] per SD, *P* = 0.013, respectively) (S10 Table). No significant associations were found for the other PRSs (S10 Table).

Second, since HDL-C was independently associated with progression to insulin in European patients [2], we replaced triglyceride and LDL-C with HDL-C in multivariate Cox analysis and confirmed its association with glycemic progression in our cohort (HR: 0.85 [95% CI 0.74–0.98], *P* = 0.025; S11 Table). We weighted the European-T2D and Asian-T2D PRS using the logarithm of the allelic odds ratio of the SNP as reported in the meta-analyses and examined the associations with glycemic progression. The results were similar to that from the unweighted PRS (HR: 1.06 [95% CI 1.01–1.11] per SD; *P* = 0.013 for the European-T2D PRS).

Third, taking into consideration of the relationship between BMI and T2D, some T2D-related SNPs may be associated with BMI. Therefore, following suggestions from reviewers, we updated the European-T2D PRS and Asian-T2D PRS by excluding those BMI-related SNPs and examined the associations between the updated PRSs and glycemic progression. We first checked for LD between the European T2D–related SNPs, or Asian T2D–related SNPs, and the list of SNPs associated with BMI using data from 1000 Genome project and data for Han Chinese population (CHB). We found that in the European T2D–related SNPs, a total of 7 SNPs (rs2867125, rs2972144, rs2307111, rs72892910, rs7903146, rs7185735, and rs12970134) had r^2^ >0.6 with any one of the BMI-related SNPs on the same chromosome. For the Asian T2D–related SNPs, we identified 4 SNPs (rs2943641, rs7903146, rs9939609, and rs12970134) that had r^2^ >0.6 with any one of the BMI SNPs on the same chromosome. After exclusion of these SNPs with high LD with BMI-related SNPs, we reconstructed the European-T2D PRS and Asian-T2D PRS and repeated the analysis. The results showed that the association between the European-T2D PRS was similar to the results of our original analysis (S12 Table). Furthermore, we identified 17 SNPs from the European T2D–related SNPs, which showed significant association at level of 0.05 with baseline BMI in our primary cohort of HKDR. The identities of these SNPs are rs780094, rs12617659, rs13389219, rs4865796, rs459193, rs10077431, rs7756992, rs853974, rs12681990, rs516946, rs10811661, rs2796441, rs2237892, rs576674, rs963740, rs982077, and rs8108269. Likewise, 11 SNPs from the list of Asian T2D–related SNPs showed association with baseline BMI (rs780094, rs3923113, rs1470579, rs459193, rs7756992, rs791595, rs7041847, rs10811661, rs2796441, rs2237892, and rs7178572). We further removed these SNPs from the respective European-T2D PRS and Asian-T2D PRS, in addition to removing the SNPs with high LD with BMI-related SNPs, and repeated the association analysis. Interestingly, the association between the European-T2D PRS and diabetes progression became stronger, whereas the results for the Asian-T2D PRS and diabetes progression remained similar, with no evidence of association (S12 Table). Replication analyses in the HKDB showed similar results (S13 Table).

Fourth, in order to explore the impact of the different drug response PRS stratified according to prescribed drugs, we investigated the associations of metformin PRS and SU PRS with glycemic progression by stratifying the exposure time of each oral drug among new users of oral drug by dividing into tertiles according to duration of drug exposure during follow-up. The interaction term between duration of metformin exposure and metformin PRS was not significantly associated with glycemic progression. However, the HR for metformin PRS increased with increasing exposure to metformin during the follow-up period, with nominal significance for the high-exposure subgroup (HR: 1.16 [95% CI 1.01–1.33]; *P* = 0.04), with adjustment for confounding factors (S14 Table). However, no significant association was observed for the metformin PRS if stratified according to duration of exposure to SU. Although the sample size was significantly reduced because of exclusion of prevalent drug users in each group and the smaller subgroups, this provides suggestive evidence that the association between metformin PRS and glycemic progression in users of metformin is mainly driven by those with longer duration of exposure to the drug.

Fifth, as the replication cohort, HKDB, included patients with a longer duration of diabetes and worse renal function compared with those in HKDR, we investigated the potential impact of CKD on the associations of PRS and glycemic progression. After excluding 688 with history of CKD, 3,251 patients from HKDB were included in this analysis (mean age 59.5 ± 10.6 [SD] years; 58.8% men; median duration of diabetes 6 [IQR: 2–12] years; median eGFR 90.2 [IQR: 78.5–98.7]). Among them, 127 (3.9%) had glycemic progression during a median follow-up period of 1.9 (IQR: 1.6–2.3) years, which was similar with the original analysis of HKDB. Analysis of different PRSs and glycemic progression in this subcohort gave similar results to that obtained from the main analysis, with only the European-T2D PRS replicated to be associated with glycemic progression (HR: 1.25 [95% CI 1.03–1.51] per SD; *P* = 0.023).

Finally, we explored potential misclassification of T2D in a subgroup of patients with available glutamic acid decarboxylase antibodies (GADA) data measured for research purpose and not for clinical indication. In this subgroup of 983 patients (91% [*N* = 895] with young-onset diabetes), 6.8% (*N* = 67) were GADA positive (≥5 μg/mL). The proportions of patients with positive GADA in different BMI categories of <18.5, 18.5–23, 23–25, and ≥25 kg/m^2^ were 19.4% (*n* = 6), 9.3% (*N* = 24), 4.9% (*N* = 9), and 5.5% (*N* = 28), respectively. Exclusion of patients with GADA did not influence the results.

## Discussion

There is considerable heterogeneity in glycemic progression in T2D. In this prospective cohort of the HKDR, we analyzed time to requirement or initiation of insulin in patients with T2D followed for a median duration of 8.8 years. Our main findings were 3-fold. First, there was an approximately 1.3-year delay of insulin use after OGLD failure in real-world practice. Second, young age of diagnosis, high HbA1c and triglyceride, extremes of BMI, use of tobacco, and microvascular complications predicted rapid glycemic progression [2,3]. Third, genetic variants associated with T2D in European populations predicted early age of diagnosis and rapid glycemic progression. These findings emphasize the importance of both genetic and modifiable risk factors, notably lipo-glucotoxicity, obesity, and tobacco use on glycemic progression, which enables identification of high-risk patients for optimal control of risk factors to improve glycemic durability.

Among progressors to insulin requirement, 33.2% experienced delay of 1.3 years before actual initiation of insulin. Compared with nonprogressors or those with more prompt insulin initiation, patients with delayed insulin treatment were younger and had shorter disease duration, lower blood pressure, and higher BMI. In a recent study from the United States, approximately 30% of patients with T2D had delayed insulin therapy, with an average lag period of 2 years [33]. In our analysis, the presence of microvascular complications was associated with earlier start of insulin, raising the possibility that delayed insulin and increased glycemic burden might contribute to increased risk of complications especially in young patients [34]. Inconvenience, fear of injection, insufficient empowerment, physicians’ concerns of patient nonadherence, and lack of supporting system might delay insulin initiation [35,36]. These barriers can be overcome by the use of team-based care with focus on education/self-care, task shifting (e.g., using nurse educator), and providing feedback to promote physician-patient communication, especially in low-resource settings [37].

It is well established that smoking is an independent risk factor for T2D [38], but our study further revealed that smoking can also influence the glycemic progression once T2D is diagnosed, suggesting that cessation of smoking is a key factor for prevention and management of T2D. Similar to reports in European populations [2], extremes of BMI predicted glycemic progression in our population, but not BMI PRS. The association of high BMI might be more related to lifestyle factors, whereas low BMI might be associated with atypical forms of diabetes due to autoimmunity or uncommon genetic variants. Indeed, in a subgroup analysis, 1 in 5 patients with BMI <18 kg/m^2^ had GADA. Although our sample size had 87% power to detect an association between BMI PRS and glycemic progression at α-level of 0.05, assuming an HR of 1.07, only 0.5% variance of BMI was explained by the PRS, comprising 63 risk variants. There are relatively few GWASs on obesity in Asians, and more BMI-related genetic variants are needed to validate the genetic association of obesity and glycemic progression.

In the IMI-DIRECT study including 5,250 patients with T2D, a high PRS derived from 61 T2D risk variants predicted young age at diagnosis but not glycemic progression [2]. In this larger cohort including over 7,000 Chinese patients with T2D, we derived a European-T2D PRS using 123 variants that predicted age at diagnosis and glycemic progression. The Asian-T2D PRS also predicted age at diagnosis but not glycemic progression. The considerably fewer Asian-T2D SNPs compared with the European-T2D SNPs might have reduced the power of the study. However, using progression to insulin as an end point in a subgroup analysis, the association with Asian-T2D PRS was validated. Given the small effect size of these common SNPs, the use of genome-wide PRS based on thousands of SNPs may further improve the discriminative performance in predicting complex traits [39]. For genetic risk of drug responses, only the metformin PRS predicted rapid glycemic progression, albeit not replicated in the HKDB cohort. We did not find any associations with SU PRS and TZD PRS. These negative associations may be due to secular changes in drug use between the primary and replication cohorts. Over 90% of patients were treated with metformin-based therapy in the primary cohort established since 1997, whereas newer drugs (e.g., dipeptidyl peptidase-4 [DPP-4] inhibitors) were more frequently prescribed in the replication cohort established since 2014. Small sample size, long disease duration, and short period of follow-up in the replication cohort may also reduce its power to detect the association. There are few pharmacogenetic studies in Asian population on OGLDs, calling for more large-scale GWASs to fill this knowledge gap.

One of the important applications from the use of such PRS in our study is to identify potentially important pathways involved in glycemic progression and generate new etiological insights. SNPs associated with T2D are most commonly associated with beta-cell function, highlighting the potential central role of beta-cell dysfunction in both the pathogenesis of T2D and glycemic progression following onset of T2D. The lack of association between the PRS for BMI and glycemic progression suggests that weight loss alone, although useful to improve metabolic control, may not have sustained effects on slowing glycemic deterioration. Recent studies in mainly European cohorts have generated phenotyped-based clusters, pathophysiology-based clusters, and PRSs that reflect different pathophysiological processes ranging from beta-cell dysfunction to adiposity to lipodystrophy [40–42]. Although beyond the scope of the current manuscript, we plan to explore the importance of these pathophysiological defects in relation to glycemic progression using these pathway-based PRSs in future analyses. The fact that over 90% of patients in HKDR were treated with metformin at some stage, and the association between metformin PRS and glycemic progression, highlights the potential utility for precision medicine in diabetes, whereby genetic determinants of response to key glucose-lowering drugs have important modulating effects on disease progression, and therefore can be used to guide treatment escalation. On the basis of findings from the current study, we have already embarked on translational studies to evaluate the use of such biomarker information to guide clinical treatment decisions.

We acknowledge several limitations in our study. First, despite excluding patients with T1D and those requiring insulin within 1 year of diagnosis, it remains possible that some patients were misdiagnosed as T2D, especially for those with low BMI. In a sensitivity analysis of a subgroup of patients, predominantly those with young-onset diabetes who had available GADA data, approximately 7% had positive GADA [43]. Although there were more patients with positive GADA in the low-BMI group compared with the other groups (19% versus 5%–10%), only 25% of patients had low BMI, and thus the impact of misclassification was only modest. However, measurement of these biomarkers, especially in young and/or lean patients, may avoid undue delay in insulin treatment. In the United Kingdom Prospective Diabetes Survey involving 3,672 patients with T2D, GADA (defined as titer >20 U/L by a radioimmunoprecipitation assay) was present in 9.8% of adult patients, who were more likely to require insulin within 6 years of diagnosis than those without GADA [44]. Second, since different definitions of phenotypes, especially for drug response, were used in previous studies together with some variants not being identified in our GWAS dataset, we did not weigh the PRSs using the reported effect sizes of these risk alleles. However, we used weighted European-T2D PRS and found similar results in the sensitivity analysis. Third, we did not have a replication cohort with detailed follow-up data of drug use as the primary cohort. However, the associations between clinical variables and end point were similar in both discovery/validation cohorts and consistent with previous studies.

In conclusion, in Chinese patients with T2D, extremes of BMI were associated with rapid glycemic progression, suggesting heterogeneous etiologies and effects of obesity in T2D. Young age at diagnosis, long disease duration, suboptimal lipid and glucose control, tobacco use, microvascular complications, and genetic variants of T2D all independently predicted glycemic progression or requirement of insulin treatment. Our study suggests potential overlap between the pathogenesis of T2D and glycemic progression. The contributions of both modifiable and nonmodifiable risk factors enable the precise identification of high-risk individuals for close monitoring and early treatment intensification to maintain glycemic durability.

## Supporting information

S1 STROBE Checklist (DOCX)

S1 Table Association of 123 European-T2D SNPs with glycemic progression. SNP, single nucleotide polymorphism; T2D, type 2 diabetes.
(DOC)

S2 Table Association of 48 Asian-T2D SNPs with glycemic progression. SNP, single nucleotide polymorphism; T2D, type 2 diabetes.
(DOC)

S3 Table Association of 63 BMI SNPs with glycemic progression. BMI, body mass index; SNP, single nucleotide polymorphism.
(DOC)

S4 Table Association of 8 metformin SNPs with glycemic progression. SNP, single nucleotide polymorphism.
(DOC)

S5 Table Association of 7 SU SNPs with glycemic progression. SNP, single nucleotide polymorphism; SU, sulphonylurea.
(DOC)

S6 Table Association of 3 TZD SNPs with glycemic progression. SNP, single nucleotide polymorphism; TZD, thiazolidinediones.
(DOC)

S7 Table Baseline characteristics of progressors and nonprogressors for progression to actual insulin treatment in the replication cohort of HKDB. HKDB, Hong Kong Diabetes Biobank.
(DOC)

S8 Table Associations of PRSs with progression to requirement of insulin treatment, age at diagnosis of diabetes, and baseline BMI in the primary cohort of HKDR. BMI, body mass index; HKDR, Hong Kong Diabetes Register; PRS, polygenic risk score.
(DOC)

S9 Table Associations of PRSs with progression to actual insulin treatment, age at diagnosis of diabetes, and baseline BMI in the replication cohort of HKDB. BMI, body mass index; HKDB, Hong Kong Diabetes Biobank; PRS, polygenic risk score.
(DOC)

S10 Table Associations of PRSs with progression to actual insulin treatment in the primary cohort of HKDR. HKDR, Hong Kong Diabetes Register; PRS, polygenic risk score.
(DOC)

S11 Table Multivariate Cox proportional hazards model with inclusion of HDL-C for diabetes progression in the primary cohort of HKDR. HDL-C, high-density lipoprotein cholesterol; HKDR, Hong Kong Diabetes Register.
(DOC)

S12 Table Associations of the European-T2D PRS and Asian-T2D PRS after excluding those BMI-related SNPs with glycemic progression in the primary cohort of HKDR. BMI, body mass index; HKDR, Hong Kong Diabetes Register; PRS, polygenic risk score; SNP, single nucleotide polymorphism; T2D, type 2 diabetes.
(DOC)

S13 Table Associations of the European-T2D PRS and Asian-T2D PRS after excluding those BMI-related SNPs with glycemic progression in the replication cohort of HKDB. BMI, body mass index; HKDB, Hong Kong Diabetes Biobank; PRS, polygenic risk score; SNP, single nucleotide polymorphism; T2D, type 2 diabetes.
(DOC)

S14 Table Associations of metformin PRS with glycemic progression, stratified by percentage of exposure time of each oral drug in HKDR. HKDR, Hong Kong Diabetes Register; PRS, polygenic risk score.
(DOC)

S1 Fig Sample selection from the discovery cohort of HKDR. HKDR, Hong Kong Diabetes Register. (TIF)

S2 Fig Sample selection from the replication cohort of HKDB. HKDB, Hong Kong Diabetes Biobank.
(TIF)

S1 Text Hong Kong Diabetes Register TRS Study Group Members. (DOC)

S2 Text Hong Kong Diabetes Biobank Study Group Members. (DOC)

## Abbreviations

ACEI: angiotensin-converting enzyme inhibitor
ACR: albumin-to-creatinine ratio
AIC: Akaike information criterion
ARB: angiotensin receptor block
BMI: body mass index
CHD: coronary heart disease
CI: confidence interval
CKD: chronic kidney disease
DPP-4: dipeptidyl peptidase-4
eGFR: estimated glomerular filtration rate
GADA: glutamic acid decarboxylase antibodies
GSA: Global Screening Array
GWAS: genome-wide association study
HDL-C: high-density lipoprotein cholesterol
HKDB: Hong Kong Diabetes Biobank
HKDR: Hong Kong Diabetes Register
HR: hazard ratio
ICD-9: *International Classification of Diseases*, *Ninth Revision*
IQR: interquartile range
LD: linkage disequilibrium
LDL-C: low-density lipoprotein cholesterol
MAF: minor allele frequency
OGLD: oral glucose-lowering drug
PRS: polygenic risk score
PVD: peripheral vascular disease
PWH: Prince of Wales Hospital
SD: standard deviation
SNP: single nucleotide polymorphism
SU: sulphonylurea
T1D: type 1 diabetes
T2D: type 2 diabetes
TZD: thiazolidinediones
WHO: World Health Organization

## References

[1] Cade WT. Diabetes-related microvascular and macrovascular diseases in the physical therapy setting. Phys Ther. 2008;88(11):1322–35. 10.2522/ptj.20080008 18801863 PMC2579903

[2] Zhou K, Donnelly LA, Morris AD, Franks PW, Jennison C, Palmer CN, et al Clinical and genetic determinants of progression of type 2 diabetes: a DIRECT study. Diabetes Care. 2014;37(3):718–24. 10.2337/dc13-1995 24186880 PMC4038744

[3] Pani LN, Nathan DM, Grant RW. Clinical predictors of disease progression and medication initiation in untreated patients with type 2 diabetes and A1C less than 7%. Diabetes Care. 2008;31(3):386–90. 10.2337/dc07-1934 18083790 PMC3829640

[4] Xue A, Wu Y, Zhu Z, Zhang F, Kemper KE, Zheng Z, et al Genome-wide association analyses identify 143 risk variants and putative regulatory mechanisms for type 2 diabetes. Nature communications. 2018;9(1):2941 10.1038/s41467-018-04951-w 30054458 PMC6063971

[5] Corbin LJ, Richmond RC, Wade KH, Burgess S, Bowden J, Smith GD, et al BMI as a Modifiable Risk Factor for Type 2 Diabetes: Refining and Understanding Causal Estimates Using Mendelian Randomization. Diabetes. 2016;65(10):3002–7. 10.2337/db16-0418 WOS:000388372900020. 27402723 PMC5279886

[6] Shikata E, Yamamoto R, Takane H, Shigemasa C, Ikeda T, Otsubo K, et al Human organic cation transporter (OCT1 and OCT2) gene polymorphisms and therapeutic effects of metformin. J Hum Genet. 2007;52(2):117–22. 10.1007/s10038-006-0087-0 . 17111267

[7] Hou W, Zhang D, Lu W, Zheng T, Wan L, Li Q, et al Polymorphism of organic cation transporter 2 improves glucose-lowering effect of metformin via influencing its pharmacokinetics in Chinese type 2 diabetic patients. Molecular diagnosis & therapy. 2015;19(1):25–33. 10.1007/s40291-014-0126-z . 25573751

[8] Jablonski KA, McAteer JB, de Bakker PI, Franks PW, Pollin TI, Hanson RL, et al Common variants in 40 genes assessed for diabetes incidence and response to metformin and lifestyle intervention in the diabetes prevention program. Diabetes. 2010;59(10):2672–81. 10.2337/db10-0543 20682687 PMC3279522

[9] Phani NM, Vohra M, Kakar A, Adhikari P, Nagri SK, D'Souza SC, et al Implication of critical pharmacokinetic gene variants on therapeutic response to metformin in Type 2 diabetes. Pharmacogenomics. 2018;19(11):905–11. 10.2217/pgs-2018-0041 WOS:000439205900005. 29914345

[10] van Leeuwen N, Nijpels G, Becker ML, Deshmukh H, Zhou K, Stricker BHC, et al A gene variant near ATM is significantly associated with metformin treatment response in type 2 diabetes: a replication and meta-analysis of five cohorts. Diabetologia. 2012;55(7):1971–7. 10.1007/s00125-012-2537-x WOS:000305215200015. 22453232 PMC3369131

[11] Zhou K, Yee SW, Seiser EL, van Leeuwen N, Tavendale R, Bennett AJ, et al Variation in the glucose transporter gene SLC2A2 is associated with glycemic response to metformin. Nat Genet. 2016 10.1038/ng.3632 . 27500523 PMC5007158

[12] Rotroff DM, Yee SW, Zhou K, Marvel SW, Shah HS, Jack JR, et al Genetic Variants in CPA6 and PRPF31 Are Associated With Variation in Response to Metformin in Individuals With Type 2 Diabetes. Diabetes. 2018;67(7):1428–40. 10.2337/db17-1164 29650774 PMC6014560

[13] Tkac I, Javorsky M, Klimcakova L, Zidzik J, Gal'a I, Babjakova E, et al A pharmacogenetic association between a variation in calpain 10 (CAPN10) gene and the response to metformin treatment in patients with type 2 diabetes. Eur J Clin Pharmacol. 2015;71(1):59–63. 10.1007/s00228-014-1774-y WOS:000347156900007. 25327507

[14] Zhou K, Donnelly L, Burch L, Tavendale R, Doney AS, Leese G, et al Loss-of-function CYP2C9 variants improve therapeutic response to sulfonylureas in type 2 diabetes: a Go-DARTS study. Clinical pharmacology and therapeutics. 2010;87(1):52–6. 10.1038/clpt.2009.176 . 19794412

[15] Xu H, Murray M, McLachlan AJ. Influence of genetic polymorphisms on the pharmacokinetics and pharmaco-dynamics of sulfonylurea drugs. Current drug metabolism. 2009;10(6):643–58. 10.2174/138920009789375388 . 19799532

[16] Shao H, Ren XM, Liu NF, Chen GM, Li WL, Zhai ZH, et al Influence of CYP2C9 and CYP2C19 genetic polymorphisms on pharmacokinetics and pharmacodynamics of gliclazide in healthy Chinese Han volunteers. Journal of clinical pharmacy and therapeutics. 2010;35(3):351–60. 10.1111/j.1365-2710.2009.01134.x WOS:000277413000011. 20831536

[17] Javorsky M, Klimcakova L, Schroner Z, Zidzik J, Babjakova E, Fabianova M, et al KCNJ11 gene E23K variant and therapeutic response to sulfonylureas. Eur J Intern Med. 2012;23(3):245–9. 10.1016/j.ejim.2011.10.018 WOS:000301125900016. 22385882

[18] Prudente S, Morini E, Lucchesi D, Lamacchia O, Bailetti D, Mercuri L, et al IRS1 G972R Missense Polymorphism Is Associated With Failure to Oral Antidiabetes Drugs in White Patients With Type 2 Diabetes From Italy. Diabetes. 2014;63(9):3135–40. 10.2337/db13-1966 WOS:000341505300031. 24947357

[19] Dawed AY, Donnelly L, Tavendale R, Carr F, Leese G, Palmer CNA, et al CYP2C8 and SLCO1B1 Variants and Therapeutic Response to Thiazolidinediones in Patients With Type 2 Diabetes. Diabetes Care. 2016;39(11):1902–8. 10.2337/dc15-2464 WOS:000386328800019. 27271184

[20] Kang ES, Park SY, Kim HJ, Kim CS, Ahn CW, Cha BS, et al Effects of Pro12Ala polymorphism of peroxisome proliferator-activated receptor gamma2 gene on rosiglitazone response in type 2 diabetes. Clinical pharmacology and therapeutics. 2005;78(2):202–8. 10.1016/j.clpt.2005.04.013 . 16084854

[21] Yang H, Ye EL, Si GX, Chen LM, Cai LQ, Ye CF, et al Adiponectin Gene Polymorphism rs2241766 T/G Is Associated with Response to Pioglitazone Treatment in Type 2 Diabetic Patients from Southern China. PLoS ONE. 2014;9(11). ARTN e112480 10.1371/journal.pone.0112480. WOS:000347121300033. 10.1371/journal.pone.0112480 PMC4236071 25405601

[22] Ma RC, Tam CH, Wang Y, Luk AO, Hu C, Yang X, et al Genetic variants of the protein kinase C-beta 1 gene and development of end-stage renal disease in patients with type 2 diabetes. JAMA: the journal of the American Medical Association. 2010;304(8):881–9. Epub 2010/08/26. 304/8/881 [pii] 10.1001/jama.2010.1191 . 20736472

[23] Jiang G, Luk AOY, Tam CHT, Xie F, Carstensen B, Lau ESH, et al Progression of diabetic kidney disease and trajectory of kidney function decline in Chinese patients with Type 2 diabetes. Kidney Int. 2019;95(1):178–87. 10.1016/j.kint.2018.08.026 . 30415941

[24] Yang XL, So WY, Kong AP, Clarke P, Ho CS, Lam CW, et al End-stage renal disease risk equations for Hong Kong Chinese patients with type 2 diabetes: Hong Kong Diabetes Registry. Diabetologia. 2006;49(10):2299–308. Epub 2006/09/01. 10.1007/s00125-006-0376-3 . 16944095

[25] Piwernetz K, Home PD, Snorgaard O, Antsiferov M, Staehr-Johansen K, Krans M. Monitoring the targets of the St Vincent Declaration and the implementation of quality management in diabetes care: the DIABCARE initiative. The DIABCARE Monitoring Group of the St Vincent Declaration Steering Committee. Diabet Med. 1993;10(4):371–7. Epub 1993/05/01. 10.1111/j.1464-5491.1993.tb00083.x . 8508624

[26] Friedewald WT, Levy RI, Fredrickson DS. Estimation of the concentration of low-density lipoprotein cholesterol in plasma, without use of the preparative ultracentrifuge. Clin Chem. 1972;18(6):499–502. Epub 1972/06/01. . 4337382

[27] Levey AS, Stevens LA, Schmid CH, Zhang YL, Castro AF 3rd, Feldman HI, et al A new equation to estimate glomerular filtration rate. Ann Intern Med. 2009;150(9):604–12. 10.7326/0003-4819-150-9-200905050-00006 19414839 PMC2763564

[28] Ma RC, Lin X, Jia W. Causes of type 2 diabetes in China. The Lancet Diabetes & endocrinology. 2014;2(12):980–91. 10.1016/S2213-8587(14)70145-7 . 25218727

[29] Locke AE, Kahali B, Berndt SI, Justice AE, Pers TH, Day FR, et al Genetic studies of body mass index yield new insights for obesity biology. Nature. 2015;518(7538):197–206. 10.1038/nature14177 25673413 PMC4382211

[30] Munthali RJ, Sahibdeen V, Kagura J, Hendry LM, Norris SA, Ong KK, et al Genetic risk score for adult body mass index associations with childhood and adolescent weight gain in an African population. Genes & nutrition. 2018;13:24 10.1186/s12263-018-0613-7 30123368 PMC6090951

[31] Grambsch PM, Therneau TM. Proportional Hazards Tests and Diagnostics Based on Weighted Residuals. Biometrika. 1994;81(3):515–26. WOS:A1994PP36700006.

[32] World Health Organization. The Asian-Pacific perspective: redefining obesity and its treatment, WHO Western Pacific Region, Geneva, Switzerland 2000.

[33] Hosomura N, Malmasi S, Timerman D, Lei VJ, Zhang H, Chang L, et al Decline of insulin therapy and delays in insulin initiation in people with uncontrolled diabetes mellitus. Diabetic Med. 2017;34(11):1599–602. 10.1111/dme.13454 WOS:000413165100014. 28905434

[34] Goodall G, Sarpong EM, Hayes C, Valentine WJ. The consequences of delaying insulin initiation in UK type 2 diabetes patients failing oral hyperglycaemic agents: a modelling study. Bmc Endocr Disord. 2009;9. Artn 19 10.1186/1472-6823-9-19. WOS:000208119200019. 10.1186/1472-6823-9-19 PMC2761913 19804622

[35] Kim SG, Kim NH, Ku BJ, Shon HS, Kim DM, Park TS, et al Delay of insulin initiation in patients with type 2 diabetes mellitus inadequately controlled with oral hypoglycemic agents (analysis of patient- and physician-related factors): A prospective observational DIPP-FACTOR study in Korea. J Diabetes Invest. 2017;8(3):346–53. 10.1111/jdi.12581 WOS:000400971000012. 27712034 PMC5415458

[36] Abu Hassan H, Tohid H, Amin RM, Bidin MBL, Muthupalaniappen L, Omar K. Factors influencing insulin acceptance among type 2 diabetes mellitus patients in a primary care clinic: a qualitative exploration. Bmc Fam Pract. 2013;14. Artn 164 10.1186/1471-2296-14-164. WOS:000327452300001. 10.1186/1471-2296-14-164 PMC4231611 24164794

[37] Lim LL, Lau ESH, Kong APS, Davies MJ, Levitt NS, Eliasson B, et al Aspects of Multicomponent Integrated Care Promote Sustained Improvement in Surrogate Clinical Outcomes: A Systematic Review and Meta-analysis. Diabetes Care. 2018;41(6):1312–20. 10.2337/dc17-2010 29784698 PMC5961399

[38] Cho YM, Kim TH, Lim S, Choi SH, Shin HD, Lee HK, et al Type 2 diabetes-associated genetic variants discovered in the recent genome-wide association studies are related to gestational diabetes mellitus in the Korean population. Diabetologia. 2009;52(2):253–61. 10.1007/s00125-008-1196-4 WOS:000262411200011. 19002430

[39] Khera AV, Chaffin M, Aragam KG, Haas ME, Roselli C, Choi SH, et al Genome-wide polygenic scores for common diseases identify individuals with risk equivalent to monogenic mutations. Nature Genetics. 2018;50(9):1219–24. 10.1038/s41588-018-0183-z WOS:000443151300011. 30104762 PMC6128408

[40] Ahlqvist E, Storm P, Karajamaki A, Martinell M, Dorkhan M, Carlsson A, et al Novel subgroups of adult-onset diabetes and their association with outcomes: a data-driven cluster analysis of six variables. The lancet Diabetes & endocrinology. 2018;6(5):361–9. Epub 2018/03/06. 10.1016/S2213-8587(18)30051-2 . 29503172

[41] Mahajan A, Wessel J, Willems SM, Zhao W, Robertson NR, Chu AY, et al Refining the accuracy of validated target identification through coding variant fine-mapping in type 2 diabetes. Nature Genetics. 2018;50(4):559–71. 10.1038/s41588-018-0084-1 WOS:000429529300016. 29632382 PMC5898373

[42] Udler MS, Kim J, von Grotthuss M, Bonas-Guarch S, Cole JB, Chiou J, et al Type 2 diabetes genetic loci informed by multi-trait associations point to disease mechanisms and subtypes: A soft clustering analysis. PLoS Med. 2018;15(9). ARTN e1002654 10.1371/journal.pmed.1002654. WOS:000445914900007. 10.1371/journal.pmed.1002654 PMC6150463 30240442

[43] Luk AO, Lau ES, Lim C, Kong AP, Chow E, Ma RC, et al Diabetes-related complications and mortality in patients with young-onset latent autoimmune diabetes: A 14-year analysis of the prospective Hong Kong Diabetes Register. Diabetes Care. 2019 6; 42(6): 1042–1050. 10.2337/dc18-1796 30967437

[44] Turner R, Stratton I, Horton V, Manley S, Zimmet P, Mackay IR, et al UKPDS 25: autoantibodies to islet-cell cytoplasm and glutamic acid decarboxylase for prediction of insulin requirement in type 2 diabetes. Lancet. 1997;350(9087):1288–93. 10.1016/s0140-6736(97)03062-6 WOS:A1997YD68900011. 9357409

