# Supplementary material for: Obesity, clinical, and genetic predictors for glycemic progression in Chinese patients with type 2 diabetes: A cohort study using the Hong Kong Diabetes Register and Hong Kong Diabetes Biobank
Source: PLoS Med. 2020 Jul 28;17(7):e1003209. doi: 10.1371/journal.pmed.1003209 (PMC7386560; doi:10.1371/journal.pmed.1003209)
Supplement: S1 Table — SNP, single nucleotide polymorphism; T2D, type 2 diabetes. (DOC) [file pmed.1003209.s002.doc]

S1 Table. Association of 123 European-T2D SNPs with glycaemic progression.

| **SNP** | **Chr** | **Position** | **Nearest gene** | **MAF** | **Risk Allele** | **Model 1  (non-adjustment)** | | **Model 2  (adjustment)** | |
| --- | --- | --- | --- | --- | --- | --- | --- | --- | --- |
|  |  |  |  |  |  | HR | P | HR | P |
| rs2296173 | 1 | 39913351 | MACF1 | 0.204 | G | 1.01 (0.94-1.09) | 0.844 | 1.04 (0.96-1.12) | 0.357 |
| rs12088739 | 1 | 51506886 | MIR4421 | 0.077 | A | 1.13 (1.01-1.27) | 0.039 | 1.16 (1.02-1.32) | 0.022 |
| rs1127655 | 1 | 117530507 | PTGFRN | 0.317 | C | 1.04 (0.98-1.11) | 0.203 | 1.1 (1.02-1.17) | 0.008 |
| rs2493394 | 1 | 120471224 | NOTCH2 | 0.037 | G | 0.98 (0.84-1.16) | 0.852 | 0.99 (0.83-1.18) | 0.888 |
| rs340874 | 1 | 214159256 | PROX1-AS1 | 0.415 | C | 1.04 (0.98-1.11) | 0.185 | 1.05 (0.98-1.12) | 0.158 |
| rs2820426 | 1 | 219660535 | LOC102723886 (LYPLAL1) | 0.382 | G | 1.04 (0.98-1.11) | 0.180 | 1.06 (0.99-1.13) | 0.093 |
| rs348330 | 1 | 229672955 | ABCB10 | 0.296 | G | 0.95 (0.89-1.01) | 0.124 | 0.92 (0.86-0.99) | 0.018 |
| rs2867125 | 2 | 622827 | TMEM18 | 0.066 | C | 0.97 (0.86-1.09) | 0.617 | 0.96 (0.84-1.09) | 0.539 |
| rs780094 | 2 | 27741237 | GCKR | 0.442 | C | 1.09 (1.03-1.16) | 0.005 | 1.09 (1.02-1.17) | 0.008 |
| rs243019 | 2 | 60585806 | MIR4432HG | 0.335 | C | 0.99 (0.93-1.06) | 0.758 | 0.98 (0.92-1.05) | 0.613 |
| rs1009358 | 2 | 65276452 | CEP68 | 0.299 | T | 0.94 (0.88-1.01) | 0.072 | 0.93 (0.87-1) | 0.061 |
| rs10169613 | 2 | 111934977 | BCL2L11 | 0.430 | C | 0.99 (0.93-1.05) | 0.627 | 0.99 (0.92-1.05) | 0.650 |
| rs12617659 | 2 | 121309759 | LOC105373585 (GLI2) | 0.204 | C | 1 (0.93-1.08) | 0.993 | 0.97 (0.9-1.05) | 0.491 |
| rs7572970 | 2 | 161136656 | RBMS1 | 0.173 | G | 1.01 (0.94-1.1) | 0.737 | 1 (0.92-1.09) | 0.988 |
| rs13389219 | 2 | 165528876 | COBLL1 | 0.073 | C | 1.03 (0.92-1.16) | 0.620 | 0.96 (0.85-1.09) | 0.540 |
| rs2972144 | 2 | 227101411 | MIR5702 | 0.071 | G | 0.96 (0.85-1.08) | 0.511 | 0.93 (0.81-1.05) | 0.232 |
| rs7561798 | 2 | 228973660 | SPHKAP | 0.328 | G | 0.99 (0.93-1.05) | 0.704 | 0.98 (0.92-1.05) | 0.613 |
| rs1899951 | 3 | 12394840 | PPARG | 0.029 | C | 1.15 (0.95-1.39) | 0.152 | 1.19 (0.97-1.46) | 0.100 |
| rs1496653 | 3 | 23454790 | UBE2E2 | 0.176 | A | 1.01 (0.93-1.09) | 0.824 | 1.01 (0.93-1.1) | 0.788 |
| rs11926707 | 3 | 46925539 | PTH1R | 0.425 | C | 0.99 (0.93-1.05) | 0.651 | 1.01 (0.95-1.08) | 0.790 |
| rs2292662 | 3 | 63897215 | ATXN7 | 0.363 | C | 1.06 (0.99-1.12) | 0.086 | 1.03 (0.97-1.11) | 0.327 |
| rs6795735 | 3 | 64705365 | ADAMTS9-AS2 | 0.297 | C | 0.97 (0.91-1.04) | 0.393 | 0.94 (0.88-1.01) | 0.118 |
| rs4472028 | 3 | 152053250 | MBNL1 | 0.394 | T | 1.03 (0.97-1.1) | 0.300 | 1.05 (0.99-1.12) | 0.120 |
| rs11925227 | 3 | 170766618 | TNIK | 0.158 | G | 0.97 (0.9-1.06) | 0.501 | 0.99 (0.9-1.08) | 0.755 |
| rs7651090 | 3 | 185513392 | IGF2BP2 | 0.252 | G | 0.98 (0.91-1.05) | 0.507 | 0.97 (0.9-1.04) | 0.388 |
| rs3887925 | 3 | 186665645 | ST6GAL1 | 0.487 | T | 1.01 (0.95-1.07) | 0.745 | 0.99 (0.93-1.06) | 0.813 |
| rs1801214 | 4 | 6303022 | WFS1 | 0.080 | T | 0.96 (0.86-1.07) | 0.415 | 0.99 (0.88-1.12) | 0.913 |
| rs17086692 | 4 | 53134293 | SPATA18 | 0.282 | G | 0.9 (0.85-0.97) | 0.003 | 0.94 (0.87-1) | 0.064 |
| rs993380 | 4 | 83584496 | SCD5 | 0.385 | A | 1.04 (0.98-1.11) | 0.181 | 1.03 (0.97-1.1) | 0.318 |
| rs7674212 | 4 | 103988899 | SLC9B2 | 0.410 | G | 1 (0.94-1.06) | 0.875 | 1.01 (0.95-1.08) | 0.735 |
| rs11098676 | 4 | 123833154 | NUDT6 | 0.042 | C | 1.12 (0.96-1.3) | 0.140 | 1.13 (0.97-1.33) | 0.119 |
| rs7685296 | 4 | 153254121 | TMEM154 | 0.442 | C | 1.03 (0.97-1.1) | 0.305 | 1.01 (0.95-1.08) | 0.680 |
| rs1061813 | 5 | 14847331 | ANKH | 0.192 | G | 0.96 (0.89-1.04) | 0.335 | 0.97 (0.89-1.05) | 0.481 |
| rs4865796 | 5 | 53272664 | ARL15 | 0.138 | A | 0.99 (0.9-1.08) | 0.795 | 1.02 (0.93-1.12) | 0.658 |
| rs459193 | 5 | 55806751 | C5orf67 | 0.494 | G | 1.01 (0.96-1.08) | 0.633 | 1.03 (0.97-1.1) | 0.353 |
| rs2307111 | 5 | 75003678 | POC5 | 0.446 | T | 1.08 (1.02-1.15) | 0.014 | 1.07 (1.01-1.15) | 0.033 |
| rs6878122 | 5 | 76427311 | ZBED3-AS1 | 0.071 | G | 1.08 (0.96-1.21) | 0.183 | 1.06 (0.94-1.2) | 0.337 |
| rs10077431 | 5 | 112927686 | YTHDC2 | 0.103 | C | 1.01 (0.91-1.11) | 0.897 | 0.96 (0.86-1.07) | 0.467 |
| rs1050226 | 6 | 7281654 | RREB1 | 0.398 | A | 0.98 (0.92-1.04) | 0.532 | 1 (0.93-1.06) | 0.896 |
| rs7756992 | 6 | 20679709 | CDKAL1 | 0.495 | G | 0.98 (0.92-1.04) | 0.433 | 0.95 (0.89-1.01) | 0.083 |
| rs2857605 | 6 | 31524851 | NFKBIL1 | 0.145 | T | 0.99 (0.91-1.07) | 0.782 | 1 (0.92-1.1) | 0.972 |
| rs1063355 | 6 | 32627714 | HLA-DQB1 | 0.361 | G | 0.99 (0.93-1.06) | 0.841 | 0.94 (0.88-1.01) | 0.083 |
| rs2071479 | 6 | 32781112 | HLA-DOB | 0.019 | T | 1 (0.81-1.25) | 0.976 | 1.06 (0.84-1.32) | 0.638 |
| rs9369425 | 6 | 43810974 | LOC107986598 (VEGFA) | 0.151 | G | 1.02 (0.94-1.11) | 0.635 | 1.05 (0.96-1.15) | 0.272 |
| rs72892910 | 6 | 50816887 | TFAP2B | 0.135 | T | 1.03 (0.95-1.13) | 0.478 | 1.08 (0.98-1.18) | 0.123 |
| rs853974 | 6 | 127068983 | RPS4XP9 | 0.466 | T | 0.99 (0.93-1.05) | 0.770 | 0.96 (0.9-1.03) | 0.240 |
| rs2246012 | 6 | 131898208 | ARG1, MED23 | 0.374 | C | 0.97 (0.92-1.04) | 0.420 | 0.99 (0.92-1.05) | 0.704 |
| rs622217 | 6 | 160766770 | SLC22A3 | 0.280 | T | 1.03 (0.96-1.1) | 0.381 | 1.06 (0.98-1.13) | 0.142 |
| rs17168486 | 7 | 14898282 | DGKB | 0.497 | T | 0.99 (0.93-1.05) | 0.680 | 1 (0.94-1.06) | 0.924 |
| rs2191348 | 7 | 15064255 | AGMO | 0.292 | T | 1 (0.94-1.07) | 0.943 | 1 (0.94-1.08) | 0.892 |
| rs2908282 | 7 | 44248828 | YKT6 | 0.181 | A | 0.98 (0.9-1.06) | 0.578 | 1.01 (0.92-1.1) | 0.874 |
| rs2299383 | 7 | 103418846 | RELN | 0.392 | T | 1.05 (0.99-1.12) | 0.102 | 1.06 (0.99-1.13) | 0.071 |
| rs13239186 | 7 | 117510621 | CTTNBP2 | 0.150 | T | 0.96 (0.88-1.04) | 0.326 | 0.91 (0.83-1) | 0.049 |
| rs13234269 | 7 | 130429186 | LOC105375508 (KLF14) | 0.304 | T | 1 (0.94-1.07) | 0.996 | 1.05 (0.98-1.13) | 0.150 |
| rs7841082 | 8 | 8168987 | SGK223 | 0.020 | C | 0.96 (0.78-1.18) | 0.682 | 0.91 (0.73-1.13) | 0.400 |
| rs11774915 | 8 | 9188762 | LOC157273(TNKS) | 0.292 | T | 1.01 (0.95-1.09) | 0.672 | 0.99 (0.92-1.06) | 0.695 |
| rs10100265 | 8 | 10633159 | PINX1 | 0.340 | A | 1.06 (0.99-1.13) | 0.082 | 1.02 (0.95-1.09) | 0.530 |
| rs17411031 | 8 | 19852310 | LPL | 0.220 | C | 1.04 (0.97-1.12) | 0.266 | 0.98 (0.91-1.06) | 0.577 |
| rs10087241 | 8 | 30863722 | PURG | 0.035 | G | 1.03 (0.88-1.22) | 0.698 | 0.97 (0.81-1.15) | 0.702 |
| rs12681990 | 8 | 36859186 | KCNU1 | 0.343 | C | 1.01 (0.95-1.08) | 0.679 | 1.04 (0.97-1.11) | 0.239 |
| rs516946 | 8 | 41519248 | ANK1 | 0.131 | C | 1.04 (0.95-1.13) | 0.448 | 1.06 (0.96-1.16) | 0.268 |
| rs7845219 | 8 | 95937502 | TP53INP1 | 0.242 | T | 1.11 (1.04-1.19) | 0.002 | 1.09 (1.01-1.17) | 0.020 |
| rs3802177 | 8 | 118185025 | SLC30A8 | 0.433 | G | 0.99 (0.94-1.06) | 0.859 | 1 (0.93-1.06) | 0.893 |
| rs2294120 | 8 | 146003567 | ZNF34 | 0.270 | A | 0.98 (0.91-1.05) | 0.495 | 0.99 (0.92-1.06) | 0.754 |
| rs10974438 | 9 | 4291928 | GLIS3 | 0.421 | C | 0.96 (0.9-1.02) | 0.197 | 0.97 (0.9-1.03) | 0.327 |
| rs1063192 | 9 | 22003367 | CDKN2B-AS1/CDKN2B | 0.173 | A | 1.07 (0.99-1.16) | 0.083 | 1.06 (0.97-1.15) | 0.182 |
| rs10811661 | 9 | 22134094 | CDKN2B-AS1 | 0.385 | T | 1.05 (0.99-1.12) | 0.127 | 1.01 (0.94-1.08) | 0.829 |
| rs1758632 | 9 | 34025640 | UBAP2 | 0.151 | G | 1.02 (0.94-1.11) | 0.623 | 0.99 (0.91-1.09) | 0.884 |
| rs17791483 | 9 | 81898980 | LOC101927450 (TLE1) | 0.041 | A | 1.04 (0.89-1.22) | 0.606 | 1.03 (0.88-1.22) | 0.692 |
| rs2796441 | 9 | 84308948 | LOC101927502 (TLE1) | 0.404 | G | 1.02 (0.96-1.09) | 0.496 | 0.99 (0.92-1.06) | 0.721 |
| rs10114341 | 9 | 96919182 | LOC107987099 (PTPDC1) | 0.108 | T | 0.99 (0.9-1.09) | 0.804 | 0.94 (0.85-1.05) | 0.270 |
| rs687621 | 9 | 136137065 | ABO | 0.387 | G | 1.03 (0.97-1.1) | 0.333 | 1.03 (0.97-1.1) | 0.321 |
| rs11257655 | 10 | 12307894 | CDC123 | 0.411 | T | 1.07 (1.01-1.14) | 0.024 | 1.09 (1.02-1.17) | 0.011 |
| rs2616132 | 10 | 71469514 | FAM241B | 0.488 | A | 0.98 (0.93-1.05) | 0.608 | 1.03 (0.96-1.1) | 0.419 |
| rs2633310 | 10 | 75594050 | CAMK2G | 0.105 | G | 0.97 (0.87-1.07) | 0.511 | 0.98 (0.88-1.09) | 0.702 |
| rs753270 | 10 | 80964975 | ZMIZ1 | 0.254 | C | 1.06 (0.99-1.14) | 0.109 | 1.04 (0.97-1.12) | 0.271 |
| rs7923866 | 10 | 94482076 | HHEX | 0.196 | C | 1.02 (0.95-1.1) | 0.611 | 1.01 (0.93-1.1) | 0.764 |
| rs11591741 | 10 | 101976501 | CHUK | 0.058 | G | 1.02 (0.9-1.16) | 0.729 | 1.22 (1.06-1.4) | 0.006 |
| rs7903146 | 10 | 114758349 | TCF7L2 | 0.031 | T | 1.01 (0.85-1.2) | 0.925 | 1.09 (0.91-1.32) | 0.338 |
| rs2421016 | 10 | 124167512 | PLEKHA1 | 0.395 | C | 1.02 (0.96-1.08) | 0.606 | 0.96 (0.9-1.02) | 0.175 |
| rs2237892 | 11 | 2839751 | KCNQ1 | 0.278 | C | 0.97 (0.91-1.04) | 0.381 | 0.96 (0.9-1.04) | 0.315 |
| rs5215 | 11 | 17408630 | KCNJ11 | 0.341 | C | 1.03 (0.97-1.1) | 0.308 | 1.02 (0.95-1.09) | 0.524 |
| rs7929543 | 11 | 49351026 | TYRL | 0.147 | C | 1.08 (0.99-1.18) | 0.066 | 1.01 (0.92-1.1) | 0.852 |
| rs1552224 | 11 | 72433098 | ARAP1 | 0.058 | A | 0.95 (0.84-1.08) | 0.445 | 0.97 (0.85-1.12) | 0.719 |
| rs10830963 | 11 | 92708710 | MTNR1B | 0.436 | G | 0.99 (0.93-1.05) | 0.757 | 1.03 (0.96-1.1) | 0.401 |
| rs7931302 | 11 | 128236058 | ETS1 | 0.149 | C | 1.02 (0.93-1.11) | 0.677 | 1.02 (0.93-1.12) | 0.627 |
| rs67232546 | 11 | 128398938 | ETS1 | 0.160 | T | 1.12 (1.04-1.22) | 0.005 | 1.12 (1.03-1.22) | 0.007 |
| rs12299509 | 12 | 4406281 | CCND2 | 0.437 | G | 0.95 (0.89-1.01) | 0.095 | 0.96 (0.9-1.03) | 0.283 |
| rs11048456 | 12 | 26463082 | ITPR2 | 0.300 | C | 1.02 (0.95-1.09) | 0.600 | 1.02 (0.95-1.09) | 0.662 |
| rs10842994 | 12 | 27965150 | LOC105369709 (KLHL42) | 0.213 | C | 0.98 (0.91-1.05) | 0.573 | 1.02 (0.94-1.1) | 0.626 |
| rs2261181 | 12 | 66212318 | RPSAP52 | 0.107 | T | 1.01 (0.91-1.11) | 0.888 | 1.01 (0.91-1.12) | 0.904 |
| rs1480474 | 12 | 66326943 | HMGA2 | 0.089 | G | 0.98 (0.88-1.09) | 0.644 | 0.96 (0.85-1.07) | 0.446 |
| rs7138300 | 12 | 71439589 | TSPAN8 | 0.353 | C | 0.98 (0.92-1.04) | 0.468 | 0.93 (0.86-0.99) | 0.027 |
| rs11107116 | 12 | 93978504 | SOCS2 | 0.294 | T | 0.99 (0.93-1.06) | 0.781 | 0.98 (0.91-1.05) | 0.540 |
| rs940904 | 12 | 123491572 | PITPNM2 | 0.141 | A | 1.05 (0.96-1.15) | 0.282 | 1.04 (0.95-1.14) | 0.429 |
| rs825476 | 12 | 124568456 | ZNF664-FAM101A | 0.353 | T | 1.02 (0.96-1.09) | 0.456 | 1.02 (0.96-1.1) | 0.517 |
| rs576674 | 13 | 33554302 | KL | 0.230 | G | 0.95 (0.88-1.02) | 0.152 | 0.98 (0.9-1.06) | 0.589 |
| rs963740 | 13 | 51096095 | DLEU1 | 0.365 | A | 0.97 (0.91-1.03) | 0.357 | 0.99 (0.93-1.06) | 0.813 |
| rs1359790 | 13 | 80717156 | LOC105370275 (SPRY2) | 0.266 | G | 1.03 (0.97-1.11) | 0.336 | 1.01 (0.94-1.09) | 0.743 |
| rs4502156 | 15 | 62383155 | C2CD4A | 0.454 | T | 0.99 (0.93-1.06) | 0.831 | 0.99 (0.93-1.06) | 0.842 |
| rs982077 | 15 | 63823301 | USP3 | 0.054 | A | 1.08 (0.94-1.24) | 0.266 | 1.13 (0.97-1.32) | 0.104 |
| rs7177055 | 15 | 77832762 | LOC101929457 (HMG20A) | 0.336 | A | 1.07 (1-1.14) | 0.044 | 1.06 (0.99-1.13) | 0.082 |
| rs4932143 | 15 | 90372067 | LOC105370965 (AP3S2) | 0.224 | G | 0.99 (0.92-1.07) | 0.835 | 1.01 (0.93-1.08) | 0.893 |
| rs9940149 | 16 | 300641 | FAM234A | 0.452 | G | 1 (0.94-1.06) | 0.975 | 1.01 (0.95-1.08) | 0.682 |
| rs7185735 | 16 | 53822651 | FTO | 0.149 | G | 1.1 (1.01-1.19) | 0.032 | 1.02 (0.93-1.11) | 0.701 |
| rs244415 | 16 | 69666683 | NFAT5 | 0.110 | G | 1.01 (0.92-1.12) | 0.814 | 1.03 (0.92-1.14) | 0.623 |
| rs77258096 | 16 | 75243772 | CTRB1 | 0.030 | C | 1.07 (0.89-1.29) | 0.456 | 1.02 (0.84-1.24) | 0.833 |
| rs2925979 | 16 | 81534790 | CMIP | 0.453 | T | 1.05 (0.99-1.11) | 0.128 | 1.06 (0.99-1.13) | 0.073 |
| rs8068804 | 17 | 3985864 | ZZEF1 | 0.159 | A | 1.06 (0.98-1.15) | 0.144 | 1.01 (0.92-1.1) | 0.874 |
| rs12945601 | 17 | 17653411 | RAI1 | 0.093 | T | 0.94 (0.85-1.04) | 0.209 | 0.99 (0.88-1.1) | 0.805 |
| rs11651755 | 17 | 36099840 | HNF1B | 0.237 | C | 1.02 (0.95-1.1) | 0.576 | 1.04 (0.96-1.12) | 0.328 |
| rs17405722 | 17 | 40542501 | STAT3 | 0.018 | A | 1.09 (0.88-1.35) | 0.445 | 1.14 (0.9-1.45) | 0.267 |
| rs9911983 | 17 | 45885756 | OSBPL7 | 0.168 | T | 1.05 (0.97-1.14) | 0.225 | 1.11 (1.02-1.21) | 0.018 |
| rs9894220 | 17 | 46989154 | UBE2Z | 0.260 | A | 1.01 (0.94-1.08) | 0.785 | 1.02 (0.95-1.1) | 0.618 |
| rs17631783 | 17 | 61687600 | TACO1 | 0.029 | C | 1.04 (0.87-1.26) | 0.649 | 1.04 (0.86-1.26) | 0.695 |
| rs7240767 | 18 | 7070642 | LAMA1 | 0.264 | C | 1.02 (0.95-1.09) | 0.601 | 1.01 (0.94-1.09) | 0.743 |
| rs12970134 | 18 | 57884750 | MC4R | 0.172 | A | 1.03 (0.95-1.12) | 0.414 | 0.98 (0.89-1.07) | 0.585 |
| rs10401969 | 19 | 19407718 | SUGP1 | 0.102 | C | 1.13 (1.03-1.25) | 0.010 | 1.1 (0.99-1.22) | 0.066 |
| rs8108269 | 19 | 46158513 | GIPR | 0.424 | G | 0.96 (0.9-1.02) | 0.207 | 0.97 (0.91-1.03) | 0.327 |
| rs6515236 | 20 | 22435749 | LOC105372562 (FOXA2) | 0.310 | A | 0.97 (0.91-1.04) | 0.382 | 0.96 (0.9-1.03) | 0.309 |
| rs6059662 | 20 | 32675727 | EIF2S2 | 0.157 | G | 1.11 (1.02-1.21) | 0.012 | 1.08 (0.99-1.19) | 0.083 |
| rs4810426 | 20 | 43001721 | HNF4A | 0.435 | T | 0.97 (0.92-1.04) | 0.401 | 0.98 (0.92-1.05) | 0.646 |
| rs4823182 | 22 | 44377442 | SAMM50 | 0.479 | G | 1 (0.94-1.06) | 0.945 | 1 (0.94-1.07) | 0.926 |

Model 2 was adjusted by all clinical risk factors identified by stepwise variable selection, including age onset of diabetes, year of diagnosis,

duration of diabetes, smoking status, strata(BMI), strata(HbA1c), log-transformed triglyceride, LDL cholesterol, log-transformed ACR, sensory neuropathy, retinopathy, history of chronic kidney disease and use of medications.
