# Supplementary material for: Obesity, clinical, and genetic predictors for glycemic progression in Chinese patients with type 2 diabetes: A cohort study using the Hong Kong Diabetes Register and Hong Kong Diabetes Biobank
Source: PLoS Med. 2020 Jul 28;17(7):e1003209. doi: 10.1371/journal.pmed.1003209 (PMC7386560; doi:10.1371/journal.pmed.1003209)
Supplement: S2 Table — SNP, single nucleotide polymorphism; T2D, type 2 diabetes. (DOC) [file pmed.1003209.s003.doc]

S2 Table. Association of 48 Asian-T2D SNPs with glycaemic progression.

| **SNP** | **Chr** | **Position** | **Nearest gene** | **MAF** | **Risk Allele** | **Model 1  (non-adjustment)** | | **Model 2  (adjustment)** | |
| --- | --- | --- | --- | --- | --- | --- | --- | --- | --- |
|  |  |  |  |  |  | HR | P | HR | P |
| rs340874 | 1 | 214159256 | PROX1-AS1 | 0.415 | C | 1.04 (0.98-1.11) | 0.185 | 1.05 (0.98-1.12) | 0.158 |
| rs780094 | 2 | 27741237 | GCKR | 0.442 | C | 1.09 (1.03-1.16) | 0.005 | 1.09 (1.02-1.17) | 0.008 |
| rs243088 | 2 | 60568745 | BCL11A | 0.335 | T | 1.02 (0.96-1.09) | 0.517 | 1.01 (0.94-1.08) | 0.751 |
| rs3923113 | 2 | 165501849 | GRB14 | 0.099 | A | 1.02 (0.92-1.12) | 0.745 | 0.99 (0.89-1.1) | 0.852 |
| rs2943641 | 2 | 227093745 | IRS1 | 0.071 | C | 0.97 (0.86-1.09) | 0.567 | 0.93 (0.82-1.05) | 0.250 |
| rs1801282 | 3 | 12393125 | PPARG | 0.029 | C | 1.16 (0.96-1.4) | 0.134 | 1.2 (0.98-1.48) | 0.079 |
| rs6780569 | 3 | 23198484 | UBE2E2 | 0.171 | G | 0.99 (0.92-1.07) | 0.844 | 0.99 (0.91-1.08) | 0.889 |
| rs831571 | 3 | 64048297 | PSMD6 | 0.359 | C | 1.07 (1.01-1.15) | 0.026 | 1.04 (0.97-1.12) | 0.241 |
| rs1470579 | 3 | 185529080 | IGFBP2 | 0.261 | C | 0.98 (0.91-1.05) | 0.566 | 0.97 (0.9-1.05) | 0.438 |
| rs6815464 | 4 | 1309901 | MAEA | 0.434 | C | 1.01 (0.95-1.08) | 0.647 | 1.05 (0.98-1.12) | 0.183 |
| rs459193 | 5 | 55806751 | C5orf67 | 0.494 | G | 1.01 (0.96-1.08) | 0.633 | 1.03 (0.97-1.1) | 0.353 |
| rs7756992 | 6 | 20679709 | CDKAL1 | 0.495 | G | 0.98 (0.92-1.04) | 0.433 | 0.95 (0.89-1.01) | 0.083 |
| rs9470794 | 6 | 38106844 | ZFAND3 | 0.362 | C | 0.96 (0.91-1.02) | 0.236 | 0.95 (0.89-1.02) | 0.162 |
| rs1535500 | 6 | 39284050 | KCNK16 | 0.499 | T | 0.96 (0.91-1.02) | 0.201 | 1 (0.94-1.06) | 0.943 |
| rs2191349 | 7 | 15064309 | DGKB | 0.293 | T | 1 (0.94-1.07) | 0.971 | 1 (0.93-1.08) | 0.925 |
| rs864745 | 7 | 28180556 | JAZF1 | 0.222 | T | 1.01 (0.94-1.09) | 0.710 | 1.02 (0.95-1.11) | 0.563 |
| rs6467136 | 7 | 127164958 | GCC1-PAX4 | 0.177 | G | 1.06 (0.98-1.15) | 0.136 | 1.05 (0.96-1.14) | 0.281 |
| rs10229583 | 7 | 127246903 | PAX4 | 0.157 | G | 1.08 (0.99-1.18) | 0.073 | 1.06 (0.97-1.16) | 0.229 |
| rs791595 | 7 | 127862802 | MIR129-LEP | 0.166 | A | 0.92 (0.85-1) | 0.046 | 1 (0.92-1.09) | 0.987 |
| rs515071 | 8 | 41519462 | ANK1 | 0.159 | G | 1.03 (0.95-1.12) | 0.444 | 1.05 (0.96-1.15) | 0.308 |
| rs896854 | 8 | 95960511 | TP53INP1 | 0.245 | T | 1.11 (1.04-1.19) | 0.003 | 1.08 (1.01-1.17) | 0.036 |
| rs13266634 | 8 | 118184783 | SLC30A8 | 0.432 | C | 0.99 (0.94-1.06) | 0.851 | 1 (0.93-1.06) | 0.904 |
| rs7041847 | 9 | 4287466 | GLIS3 | 0.490 | A | 0.96 (0.9-1.02) | 0.198 | 0.98 (0.92-1.05) | 0.642 |
| rs17584499 | 9 | 8879118 | PTPRD | 0.123 | T | 0.96 (0.87-1.05) | 0.373 | 0.99 (0.9-1.1) | 0.890 |
| rs10811661 | 9 | 22134094 | CDKN2B-AS1 | 0.385 | T | 1.05 (0.99-1.12) | 0.127 | 1.01 (0.94-1.08) | 0.829 |
| rs2796441 | 9 | 84308948 | LOC101927502 (TLE1) | 0.404 | G | 1.02 (0.96-1.09) | 0.496 | 0.99 (0.92-1.06) | 0.721 |
| rs11787792 | 9 | 139252148 | GPSM1 | 0.034 | A | 1.07 (0.9-1.28) | 0.412 | 1.04 (0.87-1.25) | 0.651 |
| rs12779790 | 10 | 12328010 | CDC123/CAMK1D | 0.207 | G | 1.06 (0.99-1.15) | 0.100 | 1.07 (0.99-1.16) | 0.102 |
| rs12571751 | 10 | 80942631 | ZMIZ1 | 0.395 | A | 1.02 (0.96-1.08) | 0.569 | 0.99 (0.93-1.06) | 0.743 |
| rs1111875 | 10 | 94462882 | HHEX/IDE | 0.305 | C | 1.03 (0.97-1.1) | 0.337 | 1.04 (0.97-1.12) | 0.268 |
| rs7903146 | 10 | 114758349 | TCF7L2 | 0.031 | T | 1.01 (0.85-1.2) | 0.925 | 1.09 (0.91-1.32) | 0.338 |
| rs10886471 | 10 | 121149403 | GRK5 | 0.195 | C | 0.99 (0.92-1.07) | 0.871 | 0.95 (0.88-1.03) | 0.233 |
| rs231362 | 11 | 2691471 | KCNQ1 | 0.090 | G | 1.06 (0.95-1.18) | 0.294 | 0.97 (0.86-1.09) | 0.571 |
| rs2237892 | 11 | 2839751 | KCNQ1 | 0.278 | C | 0.97 (0.91-1.04) | 0.381 | 0.96 (0.9-1.04) | 0.315 |
| rs5215 | 11 | 17408630 | KCNJ11 | 0.341 | C | 1.03 (0.97-1.1) | 0.308 | 1.02 (0.95-1.09) | 0.524 |
| rs1552224 | 11 | 72433098 | ARAP1 | 0.058 | A | 0.95 (0.84-1.08) | 0.445 | 0.97 (0.85-1.12) | 0.719 |
| rs1359790 | 13 | 80717156 | LOC105370275 (SPRY2) | 0.266 | G | 1.03 (0.97-1.11) | 0.336 | 1.01 (0.94-1.09) | 0.743 |
| rs7403531 | 15 | 38822905 | RASGRP1 | 0.346 | T | 0.96 (0.9-1.02) | 0.227 | 0.93 (0.87-1) | 0.054 |
| rs7172432 | 15 | 62396389 | C2CD4A-C2CD4B | 0.308 | A | 0.97 (0.91-1.04) | 0.396 | 0.98 (0.92-1.06) | 0.657 |
| rs7178572 | 15 | 77747190 | HMG20A | 0.347 | G | 1.08 (1.02-1.15) | 0.014 | 1.08 (1.01-1.16) | 0.018 |
| rs9939609 | 16 | 53820527 | FTO | 0.149 | A | 1.1 (1.01-1.19) | 0.030 | 1.02 (0.93-1.11) | 0.701 |
| rs7202877 | 16 | 75247245 | BCAR1 | 0.208 | G | 0.99 (0.92-1.07) | 0.763 | 1.01 (0.94-1.1) | 0.746 |
| rs391300 | 17 | 2216258 | SRR | 0.363 | C | 1.04 (0.97-1.1) | 0.274 | 1.02 (0.96-1.09) | 0.527 |
| rs312457 | 17 | 6940393 | SLC16A13 | 0.104 | G | 1 (0.91-1.1) | 0.996 | 0.95 (0.85-1.05) | 0.305 |
| rs4430796 | 17 | 36098040 | HNF1B (TCF2) | 0.270 | G | 0.99 (0.93-1.06) | 0.824 | 1.03 (0.96-1.11) | 0.418 |
| rs12970134 | 18 | 57884750 | MC4R | 0.172 | A | 1.03 (0.95-1.12) | 0.414 | 0.98 (0.89-1.07) | 0.585 |
| rs3786897 | 19 | 33893008 | PEPD | 0.410 | A | 1.03 (0.97-1.09) | 0.360 | 1.01 (0.95-1.08) | 0.774 |
| rs6017317 | 20 | 42946966 | FITM2-R3HDML-HNF4A | 0.422 | G | 0.99 (0.93-1.06) | 0.838 | 0.97 (0.91-1.04) | 0.407 |

Model 2 was adjusted by all clinical risk factors identified by stepwise variable selection, including age onset of diabetes, year of diagnosis,

duration of diabetes, smoking status, strata(BMI), strata(HbA1c), log-transformed triglyceride, LDL cholesterol, log-transformed ACR, sensory neuropathy, retinopathy, history of chronic kidney disease and use of medications.
