# Supplementary material for: Obesity, clinical, and genetic predictors for glycemic progression in Chinese patients with type 2 diabetes: A cohort study using the Hong Kong Diabetes Register and Hong Kong Diabetes Biobank
Source: PLoS Med. 2020 Jul 28;17(7):e1003209. doi: 10.1371/journal.pmed.1003209 (PMC7386560; doi:10.1371/journal.pmed.1003209)
Supplement: S3 Table — BMI, body mass index; SNP, single nucleotide polymorphism. (DOC) [file pmed.1003209.s004.doc]

S3 Table. Association of 63 BMI SNPs with glycaemic progression.

| **SNP** | **Chr** | **Position** | **Nearest gene** | **MAF** | **Risk Allele** | **Model 1  (non-adjustment)** | | **Model 2  (adjustment)** | |
| --- | --- | --- | --- | --- | --- | --- | --- | --- | --- |
|  |  |  |  |  |  | HR | P | HR | P |
| rs1000940 | 17 | 5283252 | RABEP1 | 0.370 | G | 1.07 (1-1.13) | 0.044 | 1.05 (0.98-1.12) | 0.136 |
| rs10408163 | 19 | 47597102 | ZC3H4 | 0.302 | T | 1 (0.94-1.07) | 0.997 | 0.97 (0.9-1.04) | 0.370 |
| rs10733682 | 9 | 129460914 | LMX1B | 0.241 | A | 1.02 (0.95-1.09) | 0.595 | 1.09 (1.01-1.18) | 0.028 |
| rs10968576 | 9 | 28414339 | LINGO2 | 0.160 | G | 1.01 (0.93-1.1) | 0.794 | 0.99 (0.91-1.08) | 0.888 |
| rs11126666 | 2 | 26928811 | KCNK3 | 0.298 | A | 0.97 (0.91-1.04) | 0.374 | 0.95 (0.89-1.02) | 0.194 |
| rs11583200 | 1 | 50559820 | ELAVL4 | 0.081 | C | 0.95 (0.85-1.06) | 0.370 | 0.96 (0.86-1.08) | 0.540 |
| rs1167827 | 7 | 75163169 | HIP1 | 0.069 | G | 0.92 (0.81-1.04) | 0.167 | 0.99 (0.87-1.12) | 0.837 |
| rs11688816 | 2 | 63053048 | EHBP1 | 0.294 | G | 1.03 (0.96-1.1) | 0.408 | 1.02 (0.95-1.1) | 0.554 |
| rs12286929 | 11 | 115022404 | CADM1 | 0.294 | G | 0.99 (0.93-1.06) | 0.879 | 1 (0.93-1.07) | 0.903 |
| rs12566985 | 1 | 75002193 | FPGT-TNNI3K | 0.170 | G | 1.01 (0.93-1.09) | 0.803 | 1.03 (0.94-1.12) | 0.554 |
| rs12940622 | 17 | 78615571 | RPTOR | 0.290 | G | 1.07 (1-1.14) | 0.061 | 1.13 (1.05-1.21) | 0.001 |
| rs13021737 | 2 | 632348 | TMEM18 | 0.067 | G | 0.97 (0.86-1.1) | 0.673 | 0.96 (0.84-1.1) | 0.557 |
| rs1441264 | 13 | 79580919 | MIR548A2 | 0.402 | A | 0.96 (0.9-1.02) | 0.155 | 0.96 (0.9-1.03) | 0.256 |
| rs1460676 | 2 | 164567689 | FIGN | 0.361 | C | 1.01 (0.95-1.07) | 0.848 | 0.99 (0.92-1.05) | 0.687 |
| rs1516725 | 3 | 185824004 | ETV5 | 0.073 | C | 0.94 (0.84-1.05) | 0.254 | 0.97 (0.86-1.09) | 0.606 |
| rs1528435 | 2 | 181550962 | UBE2E3 | 0.349 | T | 1.02 (0.96-1.09) | 0.486 | 1 (0.93-1.07) | 0.895 |
| rs1558902 | 16 | 53803574 | FTO | 0.152 | A | 1.1 (1.01-1.2) | 0.024 | 1.02 (0.93-1.11) | 0.706 |
| rs16907751 | 8 | 81375457 | ZBTB10 | 0.219 | C | 0.95 (0.89-1.03) | 0.200 | 0.94 (0.87-1.01) | 0.104 |
| rs16951275 | 15 | 68077168 | MAP2K5 | 0.412 | T | 0.96 (0.9-1.02) | 0.146 | 0.96 (0.89-1.02) | 0.180 |
| rs17001561 | 4 | 77096118 | SCARB2 | 0.024 | G | 1.07 (0.88-1.31) | 0.493 | 1 (0.81-1.24) | 0.996 |
| rs17405819 | 8 | 76806584 | HNF4G | 0.466 | T | 1.04 (0.98-1.11) | 0.160 | 1.05 (0.98-1.12) | 0.152 |
| rs17724992 | 19 | 18454825 | PGPEP1 | 0.461 | A | 1 (0.94-1.06) | 0.907 | 1 (0.94-1.07) | 0.993 |
| rs1808579 | 18 | 21104888 | C18orf8 | 0.384 | C | 1.05 (0.98-1.11) | 0.163 | 1.02 (0.96-1.09) | 0.515 |
| rs1928295 | 9 | 120378483 | TLR4 | 0.397 | T | 1.04 (0.98-1.11) | 0.209 | 1.03 (0.96-1.1) | 0.459 |
| rs2033732 | 8 | 85079709 | RALYL | 0.408 | C | 0.95 (0.89-1.01) | 0.080 | 0.96 (0.9-1.03) | 0.281 |
| rs205262 | 6 | 34563164 | C6orf106 | 0.149 | G | 0.99 (0.91-1.08) | 0.809 | 0.95 (0.87-1.05) | 0.301 |
| rs2075650 | 19 | 45395619 | TOMM40 | 0.083 | A | 1.05 (0.94-1.18) | 0.366 | 1.03 (0.91-1.17) | 0.604 |
| rs2080454 | 16 | 49062590 | CBLN1 | 0.495 | C | 0.99 (0.93-1.05) | 0.722 | 0.98 (0.92-1.05) | 0.614 |
| rs2112347 | 5 | 75015242 | POC5 | 0.447 | T | 1.08 (1.02-1.15) | 0.009 | 1.08 (1.01-1.15) | 0.019 |
| rs2176040 | 2 | 227092802 | LOC646736 | 0.071 | A | 1.04 (0.92-1.16) | 0.567 | 1.08 (0.95-1.22) | 0.250 |
| rs2176598 | 11 | 43864278 | HSD17B12 | 0.134 | T | 0.97 (0.89-1.07) | 0.567 | 1 (0.91-1.1) | 0.940 |
| rs2207139 | 6 | 50845490 | TFAP2B | 0.133 | G | 1.02 (0.94-1.11) | 0.645 | 1.06 (0.96-1.16) | 0.234 |
| rs2287019 | 19 | 46202172 | QPCTL | 0.179 | C | 0.96 (0.89-1.03) | 0.275 | 0.98 (0.9-1.06) | 0.558 |
| rs2365389 | 3 | 61236462 | FHIT | 0.143 | C | 1.03 (0.94-1.12) | 0.523 | 1.05 (0.95-1.15) | 0.332 |
| rs2820292 | 1 | 201784287 | NAV1 | 0.223 | C | 0.98 (0.91-1.05) | 0.547 | 0.95 (0.88-1.03) | 0.224 |
| rs2836754 | 21 | 40291740 | ETS2 | 0.378 | C | 1.02 (0.96-1.08) | 0.593 | 1.01 (0.95-1.08) | 0.713 |
| rs29941 | 19 | 34309532 | KCTD15 | 0.212 | G | 1.01 (0.94-1.09) | 0.819 | 0.95 (0.88-1.03) | 0.190 |
| rs3101336 | 1 | 72751185 | NEGR1 | 0.077 | C | 1 (0.89-1.12) | 0.987 | 1.01 (0.9-1.14) | 0.809 |
| rs3736485 | 15 | 51748610 | DMXL2 | 0.108 | A | 0.99 (0.9-1.09) | 0.871 | 1.02 (0.92-1.13) | 0.663 |
| rs3817334 | 11 | 47650993 | MTCH2 | 0.283 | T | 0.95 (0.89-1.02) | 0.174 | 0.95 (0.88-1.02) | 0.151 |
| rs3888190 | 16 | 28889486 | ATP2A1 | 0.090 | A | 0.99 (0.89-1.1) | 0.903 | 0.95 (0.85-1.07) | 0.420 |
| rs4256980 | 11 | 8673939 | TRIM66 | 0.402 | G | 0.96 (0.9-1.02) | 0.196 | 0.95 (0.89-1.01) | 0.100 |
| rs4740619 | 9 | 15634326 | C9orf93 | 0.244 | T | 1.03 (0.96-1.1) | 0.435 | 1.01 (0.94-1.09) | 0.811 |
| rs4787491 | 16 | 30015337 | INO80E | 0.459 | G | 1.01 (0.95-1.07) | 0.797 | 1.04 (0.97-1.11) | 0.288 |
| rs543874 | 1 | 177889480 | SEC16B | 0.148 | G | 0.96 (0.88-1.05) | 0.361 | 0.96 (0.87-1.05) | 0.362 |
| rs6465468 | 7 | 95169514 | ASB4 | 0.012 | T | 1.17 (0.89-1.53) | 0.264 | 1.17 (0.88-1.55) | 0.288 |
| rs6477694 | 9 | 111932342 | EPB41L4B | 0.433 | C | 0.98 (0.92-1.05) | 0.605 | 0.95 (0.89-1.02) | 0.143 |
| rs6567160 | 18 | 57829135 | MC4R | 0.174 | C | 1.04 (0.96-1.13) | 0.330 | 0.97 (0.89-1.06) | 0.554 |
| rs657452 | 1 | 49589847 | AGBL4 | 0.239 | A | 0.98 (0.91-1.05) | 0.552 | 0.96 (0.89-1.04) | 0.310 |
| rs6804842 | 3 | 25106437 | RARB | 0.332 | G | 1.04 (0.97-1.11) | 0.261 | 1.06 (0.98-1.13) | 0.128 |
| rs7138803 | 12 | 50247468 | BCDIN3D | 0.268 | A | 1.02 (0.95-1.09) | 0.649 | 1.03 (0.96-1.11) | 0.428 |
| rs7141420 | 14 | 79899454 | NRXN3 | 0.480 | T | 1.02 (0.96-1.08) | 0.517 | 1 (0.94-1.07) | 0.937 |
| rs7164727 | 15 | 73093991 | LOC100287559 | 0.262 | T | 1.07 (1-1.14) | 0.062 | 1.04 (0.97-1.12) | 0.295 |
| rs7239883 | 18 | 40147671 | LOC284260 | 0.317 | G | 1.01 (0.95-1.08) | 0.743 | 0.99 (0.92-1.06) | 0.694 |
| rs7243357 | 18 | 56883319 | GRP | 0.204 | T | 0.98 (0.91-1.06) | 0.638 | 0.98 (0.9-1.06) | 0.535 |
| rs7599312 | 2 | 213413231 | ERBB4 | 0.023 | G | 0.98 (0.8-1.19) | 0.821 | 1.01 (0.82-1.23) | 0.960 |
| rs7715256 | 5 | 153537893 | GALNT10 | 0.038 | G | 1.01 (0.86-1.19) | 0.888 | 1.05 (0.88-1.25) | 0.588 |
| rs7903146 | 10 | 114758349 | TCF7L2 | 0.031 | T | 1.01 (0.85-1.2) | 0.925 | 1.09 (0.91-1.32) | 0.338 |
| rs9374842 | 6 | 120185665 | LOC285762 | 0.086 | T | 1.04 (0.93-1.17) | 0.449 | 1.09 (0.97-1.22) | 0.169 |
| rs9400239 | 6 | 108977663 | FOXO3 | 0.323 | C | 1.03 (0.96-1.1) | 0.402 | 1.02 (0.95-1.09) | 0.656 |
| rs977747 | 1 | 47684677 | TAL1 | 0.047 | T | 0.94 (0.82-1.08) | 0.370 | 0.93 (0.8-1.08) | 0.333 |
| rs9914578 | 17 | 2005136 | SMG6 | 0.212 | G | 1 (0.93-1.08) | 0.920 | 1.01 (0.93-1.09) | 0.893 |
| rs9925964 | 16 | 31129895 | KAT8 | 0.120 | A | 0.95 (0.86-1.04) | 0.246 | 1.02 (0.92-1.13) | 0.695 |

Model 2 was adjusted by all clinical risk factors identified by stepwise variable selection, including age onset of diabetes, year of diagnosis, duration of diabetes, smoking status, strata(BMI), strata(HbA1c), log-transformed triglyceride, LDL cholesterol, log-transformed ACR, sensory neuropathy, retinopathy, history of chronic kidney disease and use of medications.
