# Supplementary material for: Obesity, clinical, and genetic predictors for glycemic progression in Chinese patients with type 2 diabetes: A cohort study using the Hong Kong Diabetes Register and Hong Kong Diabetes Biobank
Source: PLoS Med. 2020 Jul 28;17(7):e1003209. doi: 10.1371/journal.pmed.1003209 (PMC7386560; doi:10.1371/journal.pmed.1003209)
Supplement: S4 Table — SNP, single nucleotide polymorphism. (DOC) [file pmed.1003209.s005.doc]

S4 Table. Association of 8 metformin SNPs with glycaemic progression.

| **SNP** | **Chr** | **Position** | **Nearest gene** | **MAF** | **Risk Allele** | **Model 1  (non-adjustment)** | | **Model 2  (adjustment)** | |
| --- | --- | --- | --- | --- | --- | --- | --- | --- | --- |
|  |  |  |  |  |  | HR | P | HR | P |
| rs628031 | 6 | 160560845 | SLC22A1 | 0.270 | A | 1.07 (1-1.14) | 0.053 | 1.07 (0.99-1.15) | 0.072 |
| rs316019 | 6 | 160670282 | SLC22A2 | 0.147 | C | 0.99 (0.91-1.08) | 0.867 | 1.01 (0.92-1.1) | 0.872 |
| rs8065082 | 17 | 19465191 | SLC47A1 | 0.473 | C | 1.03 (0.97-1.09) | 0.316 | 1.06 (0.99-1.13) | 0.075 |
| rs12943590 | 17 | 19619998 | SLC47A2 | 0.461 | A | 1.01 (0.95-1.08) | 0.662 | 1.06 (0.99-1.13) | 0.092 |
| rs11212617 | 11 | 108283161 | C11orf65 | 0.366 | A | 1.01 (0.95-1.08) | 0.656 | 1.01 (0.95-1.08) | 0.724 |
| rs8192675 | 3 | 170724883 | SLC2A2 | 0.232 | T | 0.96 (0.9-1.03) | 0.297 | 0.98 (0.91-1.06) | 0.640 |
| rs57081354 | 13 | 35776594 | NBEA | 0.057 | C | 1.15 (1.01-1.3) | 0.035 | 1.15 (1-1.31) | 0.049 |
| rs3792269 | 2 | 241531479 | CAPN10 | 0.110 | G | 1.01 (0.92-1.11) | 0.829 | 1.03 (0.93-1.14) | 0.626 |

Model 2 was adjusted by all clinical risk factors identified by stepwise variable selection, including age onset of diabetes, year of diagnosis, duration of diabetes, smoking status, strata(BMI), strata(HbA1c), log-transformed triglyceride, LDL cholesterol, log-transformed ACR, sensory neuropathy, retinopathy, history of chronic kidney disease and use of medications.
