# Supplementary material for: Obesity, clinical, and genetic predictors for glycemic progression in Chinese patients with type 2 diabetes: A cohort study using the Hong Kong Diabetes Register and Hong Kong Diabetes Biobank
Source: PLoS Med. 2020 Jul 28;17(7):e1003209. doi: 10.1371/journal.pmed.1003209 (PMC7386560; doi:10.1371/journal.pmed.1003209)
Supplement: S5 Table — SNP, single nucleotide polymorphism; SU, sulphonylurea. (DOC) [file pmed.1003209.s006.doc]

S5 Table. Association of 7 SU SNPs with glycaemic progression.

| **SNP** | **Chr** | **Position** | **Nearest gene** | **MAF** | **Risk Allele** | **Model 1  (non-adjustment)** | | **Model 2  (adjustment)** | |
| --- | --- | --- | --- | --- | --- | --- | --- | --- | --- |
|  |  |  |  |  |  | HR | P | HR | P |
| rs1057910 | 10 | 96741053 | CYP2C9 | 0.031 | A | 0.99 (0.83-1.17) | 0.866 | 1.06 (0.88-1.28) | 0.544 |
| rs4244285 | 10 | 96541616 | CYP2C19 | 0.308 | A | 0.94 (0.88-1) | 0.069 | 0.95 (0.88-1.01) | 0.112 |
| rs4986893 | 10 | 96540410 | CYP2C19 | 0.055 | A | 1.05 (0.92-1.19) | 0.494 | 1.06 (0.93-1.22) | 0.389 |
| rs7903146 | 10 | 114758349 | TCF7L2 | 0.031 | T | 1.01 (0.85-1.2) | 0.925 | 1.09 (0.91-1.32) | 0.338 |
| rs757110 | 11 | 17418477 | ABCC8 | 0.357 | A | 0.98 (0.92-1.04) | 0.544 | 1 (0.93-1.07) | 0.996 |
| rs5219 | 11 | 17409572 | KCNJ11 | 0.340 | C | 0.97 (0.91-1.03) | 0.321 | 0.98 (0.91-1.05) | 0.539 |
| rs1801278 | 2 | 227660544 | IRS1 | 0.018 | C | 1.03 (0.82-1.3) | 0.775 | 1.05 (0.82-1.34) | 0.712 |

Model 2 was adjusted by all clinical risk factors identified by stepwise variable selection, including age onset of diabetes, year of diagnosis, duration of diabetes, smoking status, strata(BMI), strata(HbA1c), log-transformed triglyceride, LDL cholesterol, log-transformed ACR, sensory neuropathy, retinopathy, history of chronic kidney disease and use of medications.
