# Supplementary material for: Obesity, clinical, and genetic predictors for glycemic progression in Chinese patients with type 2 diabetes: A cohort study using the Hong Kong Diabetes Register and Hong Kong Diabetes Biobank
Source: PLoS Med. 2020 Jul 28;17(7):e1003209. doi: 10.1371/journal.pmed.1003209 (PMC7386560; doi:10.1371/journal.pmed.1003209)
Supplement: S6 Table — SNP, single nucleotide polymorphism; TZD, thiazolidinediones. (DOC) [file pmed.1003209.s007.doc]

S6 Table. Association of 3 TZD SNPs with glycaemic progression.

| **SNP** | **Chr** | **Position** | **Nearest gene** | **MAF** | **Risk Allele** | **Model 1  (non-adjustment)** | | **Model 2  (adjustment)** | |
| --- | --- | --- | --- | --- | --- | --- | --- | --- | --- |
|  |  |  |  |  |  | HR | P | HR | P |
| rs4149056 | 12 | 21331549 | SLCO1B1 | 0.129 | T | 0.99 (0.91-1.08) | 0.855 | 0.99 (0.9-1.09) | 0.800 |
| rs1801282 | 3 | 12393125 | PPARG | 0.029 | C | 1.16 (0.96-1.4) | 0.134 | 1.2 (0.98-1.48) | 0.079 |
| rs1501299 | 3 | 186571123 | ADIPOQ,ADIPOQ-AS1 | 0.268 | T | 0.99 (0.93-1.06) | 0.852 | 1.03 (0.96-1.1) | 0.460 |

Model 2 was adjusted by all clinical risk factors identified by stepwise variable selection, including age onset of diabetes, year of diagnosis, duration of diabetes, smoking status, strata(BMI), strata(HbA1c), log-transformed triglyceride, LDL cholesterol, log-transformed ACR, sensory neuropathy, retinopathy, history of chronic kidney disease and use of medications.
