# Supplementary material for: Obesity, clinical, and genetic predictors for glycemic progression in Chinese patients with type 2 diabetes: A cohort study using the Hong Kong Diabetes Register and Hong Kong Diabetes Biobank
Source: PLoS Med. 2020 Jul 28;17(7):e1003209. doi: 10.1371/journal.pmed.1003209 (PMC7386560; doi:10.1371/journal.pmed.1003209)
Supplement: S7 Table — HKDB, Hong Kong Diabetes Biobank. (DOC) [file pmed.1003209.s008.doc]

S7 Table. Baseline characteristics of progressors and non-progressors for progression to actual insulin treatment in the replication cohort of HKDB.

|  | Non-Progressors | Progressors | P |
| --- | --- | --- | --- |
| *N* | 3767 | 172 |  |
| Age (year) | 60.9 ± 10.9 | 59.7 ± 11.3 | 0.151 |
| Age at diagnosis (year) | 52.7 ± 10.7 | 46.5 ± 11.1 | <0.001 |
| Year of diagnosis | 2008 (2003-2012) | 2002 (1997-2007) | <0.001 |
| Male sex | 59.5% (2240) | 61% (105) | 0.679 |
| Duration of diabetes (year) | 7 (3-12) | 13 (7.8-18) | <0.001 |
| Smoking status |  |  | 0.106 |
| Former | 21.2% (797) | 14.5% (25) |  |
| Current | 11.6% (435) | 13.4% (23) |  |
| BMI (kg/m^2^) | 26 ± 4.4 | 26.2 ± 4.5 | 0.574 |
| HbA1c (%) | 7.2 ± 1.1 | 8.7 ± 1.8 | <0.001 |
| Triglyceride (mmol/L) | 1.3 (1-1.9) | 1.4 (0.9-2.2) | 0.03 |
| HDL cholesterol (mmol/L) | 1.2 ± 0.3 | 1.2 ± 0.3 | 0.002 |
| LDL cholesterol (mmol/L) | 2.5 ± 0.8 | 2.3 ± 0.8 | 0.074 |
| Systolic BP (mmHg) | 134.3 ± 17.6 | 135 ± 16.2 | 0.572 |
| Diastolic BP (mmHg) | 75.9 ± 11.2 | 75.3 ± 11.8 | 0.557 |
| eGFR (mL min^-1^ per 1.73 m^2^) | 86.4 (70.6-97.3) | 84.2 (58.2-97.1) | 0.03 |
| CKD history | 17.1% (643) | 26.2% (45) | 0.002 |
| Lipid lowering drugs | 65.8% (2470) | 65.1% (112) | 0.854 |

Data are expressed as mean ± standard deviation, percentage (number) or median (inter-quartile range); T-test or Mann-Whitney Rank Sum test was used for the continuous variables, and χ^2^ test was used for the categorical variables.

Abbreviations: BMI, body mass index; BP, blood pressure; HDL, high-density lipoprotein; LDL, low-density lipoprotein; eGFR, estimated glomerular filtration rate; CKD, chronic kidney disease.
