# Supplementary material for: Obesity, clinical, and genetic predictors for glycemic progression in Chinese patients with type 2 diabetes: A cohort study using the Hong Kong Diabetes Register and Hong Kong Diabetes Biobank
Source: PLoS Med. 2020 Jul 28;17(7):e1003209. doi: 10.1371/journal.pmed.1003209 (PMC7386560; doi:10.1371/journal.pmed.1003209)
Supplement: S8 Table — BMI, body mass index; HKDR, Hong Kong Diabetes Register; PRS, polygenic risk score. (DOC) [file pmed.1003209.s009.doc]

S8 Table. Associations of PRSs with progression to requirement of insulin treatment, age at diagnosis of diabetes, and baseline BMI in the primary cohort of HKDR.

|  | Model 1: Association with progression to requirement of insulin treatment | |  | Model 2: Association with age at diagnosis of diabetes | | |  | Model 3: Association with baseline BMI | | |
| --- | --- | --- | --- | --- | --- | --- | --- | --- | --- | --- |
|  | HR (95% CI) | p-value |  | Beta | SE | p-value |  | Beta | SE | p-value |
| European-T2D PRS (per SD; #SNP=123) | **1.07 (1.03-1.12)** | **0.001** |  | **-0.617** | **0.155** | **6.58×10^-5^** |  | **-0.346** | **0.057** | **1.99×10^-9^** |
| European-T2D PRS categorized as tertiles |  |  |  |  |  |  |  |  |  |  |
| Tertile 1 | **reference** | | | | | | | | | |
| Tertile 2 | **1.21 (1.04-1.42)** | **0.015** |  | **-1.534** | **0.483** | **0.001** |  | **-0.533** | **0.18** | **0.003** |
| Tertile 3 | **1.34 (1.1-1.63)** | **0.004** |  | **-2.605** | **0.643** | **5.23×10^-5^** |  | **-1.275** | **0.24** | **1.10×10^-7^** |
|  |  |  |  |  |  |  |  |  |  |  |
| Asian-T2D PRS (per SD; #SNP=48) | 1.04 (0.99-1.08) | 0.126 |  | **-0.817** | **0.154** | **1.24×10^-7^** |  | **-0.332** | **0.058** | **8.93×10^-9^** |
| Asian-T2D PRS categorized as tertiles |  |  |  |  |  |  |  |  |  |  |
| Tertile 1 | reference | | | | | | | | | |
| Tertile 2 | 1.19 (0.98-1.46) | 0.086 |  | **-1.693** | **0.611** | **0.006** |  | -0.377 | 0.229 | 0.099 |
| Tertile 3 | 1.21 (0.95-1.53) | 0.118 |  | **-3.277** | **0.749** | **1.25×10^-5^** |  | **-0.937** | **0.281** | **8.52×10^-4^** |
|  |  |  |  |  |  |  |  |  |  |  |
| BMI PRS (per SD; #SNP=63) | 1.01 (0.96-1.05) | 0.747 |  | -0.186 | 0.155 | 0.230 |  | **0.312** | **0.057** | **5.84×10^-8^** |
| BMI PRS categorized as tertiles |  |  |  |  |  |  |  |  |  |  |
| Tertile 1 | Reference | | | | | | | | | |
| Tertile 2 | 1.03 (0.88-1.2) | 0.732 |  | 0.35 | 0.518 | 0.499 |  | **0.643** | **0.193** | **8.80×10^-4^** |
| Tertile 3 | 0.94 (0.77-1.14) | 0.516 |  | -0.513 | 0.657 | 0.435 |  | **0.994** | **0.245** | **5.02×10^-5^** |
|  |  |  |  |  |  |  |  |  |  |  |
| Metformin PRS (per SD; #SNP=8) | **1.07 (1.02-1.12)** | **0.005** |  | -0.167 | 0.154 | 0.279 |  | -0.006 | 0.058 | 0.911 |
| Metformin PRS categorized as tertiles |  |  |  |  |  |  |  |  |  |  |
| Tertile 1 | Reference | | | | | | | | | |
| Tertile 2 | 1.08 (0.9-1.28) | 0.412 |  | -0.82 | 0.562 | 0.145 |  | -0.303 | 0.21 | 0.149 |
| Tertile 3 | **1.27 (1.04-1.56)** | **0.022** |  | -1.208 | 0.682 | 0.076 |  | -0.301 | 0.255 | 0.237 |
|  |  |  |  |  |  |  |  |  |  |  |
| SU PRS (per SD; #SNP=7) | 0.99 (0.94-1.04) | 0.638 |  | 0.086 | 0.153 | 0.574 |  | -0.025 | 0.057 | 0.658 |
| SU PRS categorized as tertiles |  |  |  |  |  |  |  |  |  |  |
| Tertile 1 | reference | | | | | | | | | |
| Tertile 2 | 0.93 (0.81-1.07) | 0.296 |  | 0.65 | 0.473 | 0.169 |  | -0.007 | 0.177 | 0.967 |
| Tertile 3 | 0.93 (0.8-1.09) | 0.371 |  | 0.462 | 0.529 | 0.383 |  | -0.08 | 0.198 | 0.685 |
|  |  |  |  |  |  |  |  |  |  |  |
| TZD PRS (per SD; #SNP=3) | 1.02 (0.98-1.07) | 0.345 |  | -0.175 | 0.154 | 0.256 |  | -0.021 | 0.058 | 0.710 |
| TZD PRS categorized as tertiles |  |  |  |  |  |  |  |  |  |  |
| Tertile 1 | reference | | | | | | | | | |
| Tertile 2 | 1.03 (0.69-1.53) | 0.889 |  | -1.171 | 1.199 | 0.329 |  | -0.648 | 0.448 | 0.148 |
| Tertile 3 | 1.13 (0.76-1.69) | 0.549 |  | -1.226 | 1.212 | 0.312 |  | -0.512 | 0.453 | 0.258 |
|  |  |  |  |  |  |  |  |  |  |  |
| Drug-combined PRS (per SD; #SNP=18) | 1.04 (1-1.09) | 0.062 |  | -0.114 | 0.154 | 0.459 |  | -0.028 | 0.058 | 0.625 |
| Drug-combined PRS categorized as tertiles |  |  |  |  |  |  |  |  |  |  |
| Tertile 1 | reference | | | | | | | | | |
| Tertile 2 | 1.07 (0.95-1.19) | 0.262 |  | -0.101 | 0.368 | 0.784 |  | -0.018 | 0.138 | 0.898 |
| Tertile 3 | **1.22 (1.03-1.45)** | **0.022** |  | -1.088 | 0.608 | 0.074 |  | -0.112 | 0.227 | 0.623 |

Model 1 was derived from Cox regression with adjustment for all clinical risk factors identified by stepwise variable selection.

Model 2 and Model 3 were derived from linear regression with adjustment for all potential confounding factors.

Bold highlighted represents that the association was significant at the level of 0.05.
