# Supplementary material for: Obesity, clinical, and genetic predictors for glycemic progression in Chinese patients with type 2 diabetes: A cohort study using the Hong Kong Diabetes Register and Hong Kong Diabetes Biobank
Source: PLoS Med. 2020 Jul 28;17(7):e1003209. doi: 10.1371/journal.pmed.1003209 (PMC7386560; doi:10.1371/journal.pmed.1003209)
Supplement: S9 Table — BMI, body mass index; HKDB, Hong Kong Diabetes Biobank; PRS, polygenic risk score. (DOC) [file pmed.1003209.s010.doc]

S9 Table. Associations of PRSs with progression to actual insulin treatment, age at diagnosis of diabetes, and baseline BMI in the replication cohort of HKDB.

|  | Model 1:  Association with progression to insulin treatment | |  | Model 2: Association with age at diagnosis of diabetes | | |  | Model 3: Association with baseline BMI | | |
| --- | --- | --- | --- | --- | --- | --- | --- | --- | --- | --- |
|  | HR | p-value |  | Beta | SE | p-value |  | Beta | SE | p-value |
| European-T2D GRS (per SD; #SNP=122) | 1.24 (1.06-1.46) | 0.008 |  | -0.658 | 0.15 | 1.15×10^-5^ |  | -0.482 | 0.074 | 6.71×10^-11^ |
| European-T2D GRS categorized as tertiles |  |  |  |  |  |  |  |  |  |  |
| Tertile 1 | reference | | | | | | | | | |
| Tertile 2 | 1.77 (0.98-3.22) | 0.060 |  | -1.113 | 0.442 | 0.012 |  | -0.85 | 0.217 | 9.26×10^-5^ |
| Tertile 3 | 2.52 (1.26-5.03) | 0.009 |  | -1.997 | 0.62 | 0.001 |  | -1.957 | 0.304 | 1.33×10^-10^ |
|  |  |  |  |  |  |  |  |  |  |  |
| Asian-T2D GRS (per SD; #SNP=48) | 1.09 (0.93-1.28) | 0.306 |  | -0.704 | 0.152 | 3.94×10^-6^ |  | -0.281 | 0.076 | 2.03×10^-4^ |
| Asian-T2D GRS categorized as tertiles |  |  |  |  |  |  |  |  |  |  |
| Tertile 1 | reference | | | | | | | | | |
| Tertile 2 | 1.15 (0.68-1.96) | 0.593 |  | -0.992 | 0.459 | 0.0310 |  | -0.374 | 0.228 | 0.100 |
| Tertile 3 | 1.48 (0.78-2.83) | 0.233 |  | -1.956 | 0.643 | 0.0024 |  | -0.704 | 0.319 | 0.027 |
|  |  |  |  |  |  |  |  |  |  |  |
| BMI GRS (per SD; #SNP=63) | 1.03 (0.88-1.2) | 0.693 |  | -0.259 | 0.149 | 0.0818 |  | 0.24 | 0.073 | 0.001 |
| BMI GRS categorized as tertiles |  |  |  |  |  |  |  |  |  |  |
| Tertile 1 |  | |  |  |  |  |  |  |  |  |
| Tertile 2 | 0.89 (0.53-1.51) | 0.674 |  | -1.285 | 0.508 | 0.011 |  | 0.565 | 0.251 | 0.025 |
| Tertile 3 | 0.81 (0.42-1.55) | 0.522 |  | -1.658 | 0.626 | 0.008 |  | 0.68 | 0.31 | 0.028 |
|  |  |  |  |  |  |  |  |  |  |  |
| Metformin GRS (per SD; #SNP=8) | 0.9 (0.77-1.05) | 0.172 |  | 0.008 | 0.148 | 0.959 |  | -0.002 | 0.073 | 0.980 |
| Metformin GRS categorized as tertiles |  |  |  |  |  |  |  |  |  |  |
| Tertile 1 | reference | | | | | | | | | |
| Tertile 2 | 0.58 (0.36-0.94) | 0.026 |  | 0.423 | 0.52 | 0.416 |  | 0.138 | 0.257 | 0.593 |
| Tertile 3 | 0.64 (0.35-1.16) | 0.140 |  | 0.096 | 0.644 | 0.881 |  | 0.202 | 0.319 | 0.526 |
|  |  |  |  |  |  |  |  |  |  |  |
| SU GRS (per SD; #SNP=7) | 0.99 (0.85-1.16) | 0.925 |  | 0.172 | 0.15 | 0.250 |  | -0.045 | 0.074 | 0.543 |
| SU GRS categorized as tertiles |  |  |  |  |  |  |  |  |  |  |
| Tertile 1 | reference | | | | | | | | | |
| Tertile 2 | 0.9 (0.57-1.43) | 0.657 |  | 0.765 | 0.451 | 0.089 |  | -0.343 | 0.223 | 0.124 |
| Tertile 3 | 1.01 (0.6-1.7) | 0.977 |  | 0.638 | 0.513 | 0.214 |  | -0.305 | 0.254 | 0.230 |
|  |  |  |  |  |  |  |  |  |  |  |
| TZD GRS (per SD; #SNP=3) | 1.04 (0.89-1.21) | 0.640 |  | 0.015 | 0.149 | 0.918 |  | -0.137 | 0.074 | 0.062 |
| TZD GRS categorized as tertiles |  |  |  |  |  |  |  |  |  |  |
| Tertile 1 | reference | | | | | | | | | |
| Tertile 2 | 2.67 (0.37-19.27) | 0.330 |  | -2.294 | 1.084 | 0.034 |  | -0.458 | 0.537 | 0.393 |
| Tertile 3 | 2.99 (0.41-21.68) | 0.279 |  | -1.961 | 1.097 | 0.074 |  | -0.529 | 0.543 | 0.330 |
|  |  |  |  |  |  |  |  |  |  |  |
| Drug-combined GRS (per SD; #SNP=18) | 0.93 (0.8-1.09) | 0.380 |  | 0.144 | 0.15 | 0.336 |  | -0.064 | 0.074 | 0.385 |
| Drug-combined GRS categorized as tertiles |  |  |  |  |  |  |  |  |  |  |
| Tertile 1 | reference | | | | | | | | | |
| Tertile 2 | 0.79 (0.43-1.44) | 0.441 |  | 1.255 | 0.611 | 0.040 |  | 0.129 | 0.303 | 0.669 |
| Tertile 3 | 0.71 (0.36-1.4) | 0.320 |  | 1.315 | 0.692 | 0.058 |  | -0.149 | 0.343 | 0.665 |

Model 1 was adjusted for age at diagnosis, sex, year of diagnosis, duration of diabetes, smoking status, log-transformed triglyceride, eGFR, use of lipid lowering drugs, history of CKD, and strata variables of HbA1c and BMI categories.

Model 2 was adjusted for sex, year of diagnosis, duration of diabetes, smoking status, log-transformed triglyceride, eGFR, use of lipid lowering drugs, history of CKD, HbA1c and BMI.

Model 3 was adjusted for age at diagnosis, sex, year of diagnosis, duration of diabetes, smoking status, log-transformed triglyceride, eGFR, use of lipid lowering drugs, history of CKD, and HbA1c.
