# Supplementary material for: Obesity, clinical, and genetic predictors for glycemic progression in Chinese patients with type 2 diabetes: A cohort study using the Hong Kong Diabetes Register and Hong Kong Diabetes Biobank
Source: PLoS Med. 2020 Jul 28;17(7):e1003209. doi: 10.1371/journal.pmed.1003209 (PMC7386560; doi:10.1371/journal.pmed.1003209)
Supplement: S10 Table — HKDR, Hong Kong Diabetes Register; PRS, polygenic risk score. (DOC) [file pmed.1003209.s011.doc]

S10 Table. Associations of PRSs with progression to actual insulin treatment in the primary cohort of HKDR.

|  | Model 1 (Non-adjustment) | |  | Model 2 (Adjustment for confounding factors) | |
| --- | --- | --- | --- | --- | --- |
|  |  |  |  |  |  |
|  | HR | p-value |  | HR | p-value |
| European-T2D PRS (per SD; #SNP=123) | **1.07 (1.03-1.12)** | **0.002** |  | **1.07 (1.02-1.12)** | **0.004** |
| European-T2D PRS categorized as tertiles |  |  |  |  |  |
| Tertile 1 | Reference | | | | |
| Tertile 2 | **1.23 (1.06-1.43)** | **0.008** |  | **1.21 (1.03-1.42)** | **0.024** |
| Tertile 3 | **1.4 (1.16-1.69)** | **0.001** |  | **1.34 (1.09-1.64)** | **0.005** |
|  |  |  |  |  |  |
| Asian-T2D PRS (per SD; #SNP=48) | **1.05 (1-1.1)** | **0.035** |  | 1.04 (0.99-1.09) | 0.119 |
| Asian-T2D PRS categorized as tertiles |  |  |  |  |  |
| Tertile 1 | Reference | | | | |
| Tertile 2 | 1.09 (0.9-1.3) | 0.383 |  | 1.02 (0.91-1.15) | 0.688 |
| Tertile 3 | 1.19 (0.96-1.48) | 0.113 |  | **1.21 (1.01-1.44)** | **0.039** |
|  |  |  |  |  |  |
| BMI PRS (per SD; #SNP=63) | 1.04 (1-1.09) | 0.082 |  | 1.02 (0.98-1.07) | 0.320 |
| BMI PRS categorized as tertiles |  |  |  |  |  |
| Tertile 1 | Reference | | | | |
| Tertile 2 | 1.13 (0.97-1.32) | 0.12 |  | 1.05 (0.89-1.24) | 0.571 |
| Tertile 3 | 1.03 (0.84-1.25) | 0.798 |  | 0.95 (0.77-1.17) | 0.627 |
|  |  |  |  |  |  |
| Metformin PRS (per SD; #SNP=8) | 1.04 (0.99-1.08) | 0.112 |  | **1.06 (1.01-1.11)** | **0.013** |
| Metformin PRS categorized as tertiles |  |  |  |  |  |
| Tertile 1 | Reference | | | | |
| Tertile 2 | 1.07 (0.91-1.27) | 0.401 |  | 1.06 (0.88-1.27) | 0.553 |
| Tertile 3 | 1.19 (0.97-1.45) | 0.093 |  | 1.22 (0.98-1.51) | 0.071 |
|  |  |  |  |  |  |
| SU PRS (per SD; #SNP=7) | 0.97 (0.93-1.01) | 0.174 |  | 0.98 (0.94-1.03) | 0.462 |
| SU PRS categorized as tertiles |  |  |  |  |  |
| Tertile 1 | Reference | | | | |
| Tertile 2 | 0.92 (0.8-1.05) | 0.197 |  | 0.98 (0.85-1.13) | 0.747 |
| Tertile 3 | 0.91 (0.78-1.06) | 0.224 |  | 0.95 (0.81-1.12) | 0.538 |
|  |  |  |  |  |  |
| TZD PRS (per SD; #SNP=3) | 1.01 (0.97-1.06) | 0.683 |  | 1.03 (0.98-1.08) | 0.220 |
| TZD PRS categorized as tertiles |  |  |  |  |  |
| Tertile 1 | Reference | | | | |
| Tertile 2 | 1.3 (0.88-1.91) | 0.189 |  | 1.03 (0.68-1.57) | 0.873 |
| Tertile 3 | 1.35 (0.91-1.99) | 0.135 |  | 1.17 (0.77-1.78) | 0.456 |
|  |  |  |  |  |  |
| Drug-combined PRS (per SD; #SNP=18) | 1.01 (0.96-1.05) | 0.757 |  | 1.04 (0.99-1.09) | 0.119 |
| Drug-combined PRS categorized as tertiles |  |  |  |  |  |
| Tertile 1 | Reference | | | | |
| Tertile 2 | 1.02 (0.91-1.13) | 0.774 |  | 1.02 (0.91-1.15) | 0.688 |
| Tertile 3 | 1.06 (0.9-1.26) | 0.47 |  | **1.21 (1.01-1.44)** | **0.039** |

Model 2 was derived from Cox regression with adjustment for all clinical risk factors identified by stepwise variable selection.

Bold highlighted represents that the association was significant at the level of 0.05.
