# Supplementary material for: Obesity, clinical, and genetic predictors for glycemic progression in Chinese patients with type 2 diabetes: A cohort study using the Hong Kong Diabetes Register and Hong Kong Diabetes Biobank
Source: PLoS Med. 2020 Jul 28;17(7):e1003209. doi: 10.1371/journal.pmed.1003209 (PMC7386560; doi:10.1371/journal.pmed.1003209)
Supplement: S11 Table — HDL-C, high-density lipoprotein cholesterol; HKDR, Hong Kong Diabetes Register. (DOC) [file pmed.1003209.s012.doc]

S11 Table. Multivariate Cox proportional hazards model with inclusion of HDL cholesterol for diabetes progression in the primary cohort of HKDR.

| Covariate | HR (95% CI) | P |
| --- | --- | --- |
| Age at diagnosis (per 1 year) | 0.98 (0.97-0.98) | <0.001 |
| Year of diagnosis (per 1 year) | 1.04 (1.02-1.05) | <0.001 |
| Duration of diabetes (per 1 year) | 1.06 (1.04-1.08) | <0.001 |
| Smoking |  |  |
| Ex-smoker | 1.35 (1.19-1.53) | <0.001 |
| Current smoker | 1.11 (0.98-1.27) | 0.109 |
| HDL-C | 0.85 (0.74-0.98) | 0.025 |
| log urinary ACR | 1.15 (1.11-1.19) | <0.001 |
| eGFR | 0.998 (0.996-1.001) | 0.109 |
| Sensory neuropathy | 1.27 (1.14-1.42) | <0.001 |
| Retinopathy | 1.26 (1.12-1.41) | <0.001 |
| CKD history | 1.69 (1.41-2.03) | <0.001 |
| Use of lipid-lowering drugs | 0.93 (0.8-1.07) | 0.303 |
| Use of ACEIs or ARBs | 1.11 (0.97-1.26) | 0.123 |
| Use of oral glucose lowering drugs | 1.33 (1.2-1.49) | <0.001 |

BMI and baseline HbA1c categories were included as strata variables. BMI was categorized as 4 groups (<18.5, 18.5-23, 23-25 and ≥25 kg/m2) and baseline HbA1c was categorized as 3 groups (<7%, ≥ 7-9% and ≥ 9%).
