# Supplementary material for: Obesity, clinical, and genetic predictors for glycemic progression in Chinese patients with type 2 diabetes: A cohort study using the Hong Kong Diabetes Register and Hong Kong Diabetes Biobank
Source: PLoS Med. 2020 Jul 28;17(7):e1003209. doi: 10.1371/journal.pmed.1003209 (PMC7386560; doi:10.1371/journal.pmed.1003209)
Supplement: S12 Table — BMI, body mass index; HKDR, Hong Kong Diabetes Register; PRS, polygenic risk score; SNP, single nucleotide polymorphism; T2D, type 2 diabetes. (DOC) [file pmed.1003209.s013.doc]

S12 Table. Associations of the European-T2D PRS and Asian-T2D PRS after excluding those BMI-related SNPs with glycaemic progression in the primary cohort of HKDR.

|  |  | Model 1 (Non-adjustment) | |  | Model 2 (Adjustment for confounding factors) | |
| --- | --- | --- | --- | --- | --- | --- |
|  |  | HR (95% CI) | p-value |  | HR (95% CI) | p-value |
| European-T2D PRS after  removing 7 SNPs with  high LD with BMI-related SNPs | Per SD (#SNP=116) | 1.06 (1.02-1.11) | 0.005 |  | 1.06 (1.01-1.11) | 0.013 |
|  | Categorized as tertiles |  |  |  |  |  |
|  | Tertile 1 | reference | | | | |
|  | Tertile 2 | 1.22 (1.04-1.42) | 0.013 |  | 1.17 (0.99-1.38) | 0.07 |
|  | Tertile 3 | 1.34 (1.12-1.6) | 0.001 |  | 1.26 (1.04-1.53) | 0.02 |
|  |  |  |  |  |  |  |
| European-T2D PRS after  further removing 17 SNPs with  association with baseline BMI in HKDR | Per SD (#SNP=99) | 1.06 (1.02-1.11) | 0.005 |  | 1.07 (1.02-1.12) | 0.006 |
|  | Categorized as tertiles |  |  |  |  |  |
|  | Tertile 1 | reference | | | | |
|  | Tertile 2 | 1.29 (1.11-1.5) | <0.001 |  | 1.31 (1.11-1.54) | 0.001 |
|  | Tertile 3 | 1.36 (1.14-1.62) | <0.001 |  | 1.51 (1.25-1.83) | <0.001 |
|  |  |  |  |  |  |  |
| Asian-T2D PRS after  removing 4 SNPs with  high LD with BMI-related SNPs | Per SD (#SNP=44) | 1.06 (1.01-1.1) | 0.010 |  | 1.04 (0.99-1.09) | 0.104 |
|  | Categorized as tertiles |  |  |  |  |  |
|  | Tertile 1 | reference | | | | |
|  | Tertile 2 | 1.23 (1-1.51) | 0.053 |  | 1.24 (0.99-1.55) | 0.067 |
|  | Tertile 3 | 1.25 (1-1.56) | 0.055 |  | 1.15 (0.9-1.47) | 0.256 |
|  |  |  |  |  |  |  |
| Asian-T2D PRS after  further removing 11 SNPs with  association with baseline BMI in HKDR | Per SD (#SNP=33) | 1.06 (1.01-1.1) | 0.014 |  | 1.04 (0.99-1.08) | 0.122 |
|  | Categorized as tertiles |  |  |  |  |  |
|  | Tertile 1 | reference | | | | |
|  | Tertile 2 | 0.98 (0.82-1.18) | 0.842 |  | 1.05 (0.86-1.28) | 0.619 |
|  | Tertile 3 | 1.04 (0.85-1.27) | 0.682 |  | 1.08 (0.88-1.34) | 0.464 |
