# Supplementary material for: Obesity, clinical, and genetic predictors for glycemic progression in Chinese patients with type 2 diabetes: A cohort study using the Hong Kong Diabetes Register and Hong Kong Diabetes Biobank
Source: PLoS Med. 2020 Jul 28;17(7):e1003209. doi: 10.1371/journal.pmed.1003209 (PMC7386560; doi:10.1371/journal.pmed.1003209)
Supplement: S13 Table — BMI, body mass index; HKDB, Hong Kong Diabetes Biobank; PRS, polygenic risk score; SNP, single nucleotide polymorphism; T2D, type 2 diabetes. (DOC) [file pmed.1003209.s014.doc]

S13 Table. Associations of the European-T2D PRS and Asian-T2D PRS after excluding those BMI-related SNPs with glycaemic progression in the replication cohort of HKDB.

|  |  | Model 1 (Non-adjustment) | |  | Model 2 (Adjustment for confounding factors) | |
| --- | --- | --- | --- | --- | --- | --- |
|  |  | HR (95% CI) | p-value |  | HR (95% CI) | p-value |
| European-T2D PRS after  removing 7 SNPs with  high LD with BMI-related SNPs | Per SD (#SNP=116) | 1.24 (1.06-1.44) | 0.006 |  | 1.23 (1.05-1.45) | 0.011 |
|  | Categorized as tertiles | |  |  |  |  |
|  | Tertile 1 | reference | | | | |
|  | Tertile 2 | 1.33 (0.83-2.13) | 0.236 |  | 1.42 (0.85-2.37) | 0.181 |
|  | Tertile 3 | 1.9 (1.03-3.5) | 0.040 |  | 2.17 (1.13-4.16) | 0.020 |
|  |  |  |  |  |  |  |
| European-T2D PRS after  further removing 17 SNPs with  association with baseline BMI in HKDR | Per SD (#SNP=99) | 1.22 (1.05-1.42) | 0.008 |  | 1.2 (1.03-1.41) | 0.023 |
|  | Categorized as tertiles | |  |  |  |  |
|  | Tertile 1 | reference | | | | |
|  | Tertile 2 | 1.43 (0.84-2.45) | 0.187 |  | 1.3 (0.75-2.28) | 0.353 |
|  | Tertile 3 | 2.09 (1.14-3.85) | 0.018 |  | 1.92 (1.02-3.64) | 0.045 |
|  |  |  |  |  |  |  |
| Asian-T2D PRS after  removing 4 SNPs with  high LD with BMI-related SNPs | Per SD (#SNP=44) | 1.13 (0.97-1.31) | 0.125 |  | 1.08 (0.92-1.26) | 0.371 |
|  | Categorized as tertiles | |  |  |  |  |
|  | Tertile 1 | reference | | | | |
|  | Tertile 2 | 1.06 (0.62-1.81) | 0.829 |  | 0.98 (0.57-1.69) | 0.934 |
|  | Tertile 3 | 1.6 (0.85-3) | 0.146 |  | 1.36 (0.72-2.59) | 0.346 |
|  |  |  |  |  |  |  |
| Asian-T2D PRS after  further removing 11 SNPs with  association with baseline BMI in HKDR | Per SD (#SNP=33) | 1.05 (0.91-1.23) | 0.494 |  | 1 (0.85-1.16) | 0.962 |
|  | Categorized as tertiles | |  |  |  |  |
|  | Tertile 1 | reference | | | | |
|  | Tertile 2 | 1.39 (0.77-2.51) | 0.272 |  | 1.25 (0.69-2.28) | 0.461 |
|  | Tertile 3 | 1.58 (0.81-3.11) | 0.183 |  | 1.36 (0.69-2.68) | 0.380 |
