# Supplementary material for: Obesity, clinical, and genetic predictors for glycemic progression in Chinese patients with type 2 diabetes: A cohort study using the Hong Kong Diabetes Register and Hong Kong Diabetes Biobank
Source: PLoS Med. 2020 Jul 28;17(7):e1003209. doi: 10.1371/journal.pmed.1003209 (PMC7386560; doi:10.1371/journal.pmed.1003209)
Supplement: S14 Table — HKDR, Hong Kong Diabetes Register; PRS, polygenic risk score. (DOC) [file pmed.1003209.s015.doc]

S14. Table Associations of metformin PRS with glycaemic progression stratified by percentage of exposure time of each oral drug in HKDR.

|  | Percentage of exposure time of metformin for new users of metformin | | | | | | | |
| --- | --- | --- | --- | --- | --- | --- | --- | --- |
|  | Tertile 1 (0-35%; N=1441) |  |  | Tertile 2  (35.1%-65.5%; N=616) |  |  | Tertile 3  (65.6%-99.8%; N=602) |  |
|  | HR (95% CI) | P |  | HR (95% CI) | P |  | HR (95% CI) | P |
| Metformin PRS (per SD; #SNP=8) | 1.03 (0.94-1.13) | 0.504 |  | 1.06 (0.94-1.19) | 0.356 |  | **1.16 (1.01-1.33)** | **0.040** |
| Metformin PRS categorized as tertiles |  |  |  |  |  |  |  |  |
| Tertile 1 | Ref. | / |  | Ref. | / |  | Ref. | / |
| Tertile 2 | 0.98 (0.68-1.41) | 0.919 |  | 1.39 (0.88-2.19) | 0.159 |  | 1.02 (0.61-1.68) | 0.952 |
| Tertile 3 | 1.07 (0.7-1.63) | 0.754 |  | 1.41 (0.82-2.4) | 0.212 |  | 1.56 (0.86-2.84) | 0.145 |
|  |  |  |  |  |  |  |  |  |
|  | Percentage of exposure time of SU for new users of SU | | | | | | | |
|  | Tertile 1 (0-36.3%; N=1259) |  |  | Tertile 2  (36.4%-68.1%; N=512) |  |  | Tertile 3  (68.1%-99.6%; N=512) |  |
|  | HR (95% CI) | P |  | HR (95% CI) | P |  | HR (95% CI) | P |
| Metformin PRS (per SD; #SNP=8) | 0.97 (0.87-1.08) | 0.563 |  | 1.02 (0.88-1.17) | 0.834 |  | 1.07 (0.94-1.21) | 0.334 |
| Metformin PRS categorized as tertiles |  |  |  |  |  |  |  |  |
| Tertile 1 | Ref. | / |  | Ref. | / |  | Ref. | / |
| Tertile 2 | 0.88 (0.64-1.2) | 0.422 |  | 1.05 (0.7-1.58) | 0.809 |  | 0.92 (0.59-1.45) | 0.719 |
| Tertile 3 | 0.88 (0.62-1.26) | 0.482 |  | 0.93 (0.57-1.51) | 0.773 |  | 1.23 (0.72-2.1) | 0.459 |
|  |  |  |  |  |  |  |  |  |
|  | Percentage of exposure time of TZD for new users of TZD | | | | | | | |
|  | No use of TZD (0%; N=4230) |  |  | With use of TZD  (0.2% - 94.1%; N=184) |  |  |  |  |
|  | HR (95% CI) | P |  | HR (95% CI) | P |  |  |  |
| Metformin PRS (per SD; #SNP=8) | 1.02 (0.98-1.07) | 0.312 |  | 0.98 (0.78-1.23) | 0.849 |  |  |  |
| Metformin PRS categorized as tertiles |  |  |  |  |  |  |  |  |
| Tertile 1 | Ref. | / |  | Ref. | / |  |  |  |
| Tertile 2 | 1.05 (0.7-1.58) | 0.811 |  | 0.73 (0.31-1.69) | 0.458 |  |  |  |
| Tertile 3 | 1.14 (0.76-1.72) | 0.519 |  | 1.62 (0.63-4.15) | 0.314 |  |  |  |

The percentages in parentheses represent the percentages of exposure time of each oral drug. HR and p-value were adjusted for all clinical risk factors identified by stepwise variable selection.
