# Supplementary figures and images for: Obesity, clinical, and genetic predictors for glycemic progression in Chinese patients with type 2 diabetes: A cohort study using the Hong Kong Diabetes Register and Hong Kong Diabetes Biobank
Source: PLoS Med. 2020 Jul 28;17(7):e1003209. doi: 10.1371/journal.pmed.1003209 (PMC7386560; doi:10.1371/journal.pmed.1003209)

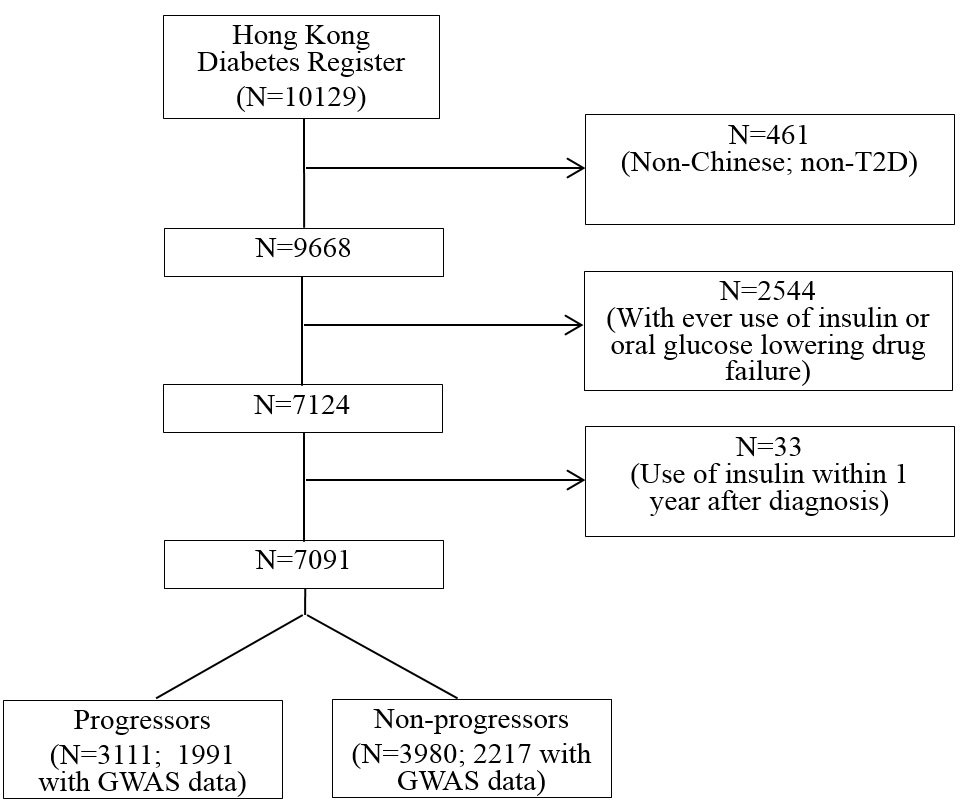

Supplement: S1 Fig — (TIF) [file pmed.1003209.s016.tif]

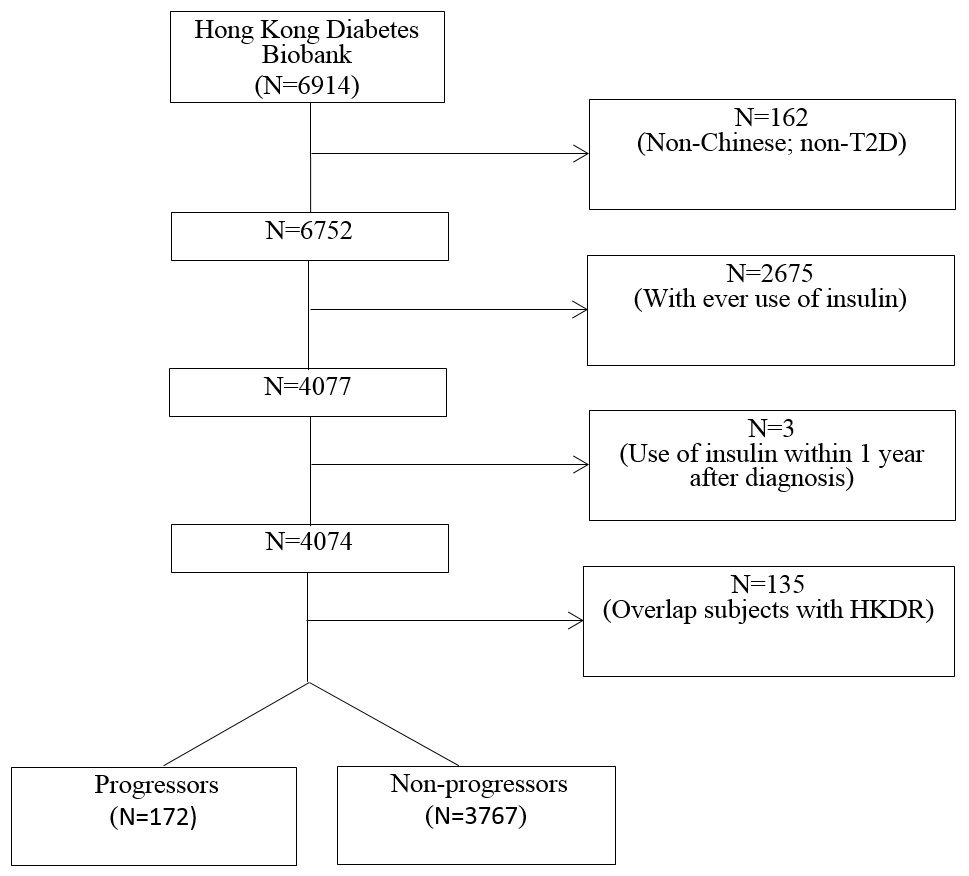

Supplement: S2 Fig — HKDB, Hong Kong Diabetes Biobank. (TIF) [file pmed.1003209.s017.tif]
