# Supplementary material for: Obesity, clinical, and genetic predictors for glycemic progression in Chinese patients with type 2 diabetes: A cohort study using the Hong Kong Diabetes Register and Hong Kong Diabetes Biobank
Source: PLoS Med. 2020 Jul 28;17(7):e1003209. doi: 10.1371/journal.pmed.1003209 (PMC7386560; doi:10.1371/journal.pmed.1003209)
Supplement: S1 Text — (DOC) [file pmed.1003209.s018.doc]

S1 Text. Hong Kong Diabetes Register TRS Study Group Members.

| Ronald C.W. Ma^1,2,3,4^  Juliana C.N. Chan^1,2,3^  Yu Huang^5^  Hui-yao Lan^1, 3^  Si Lok ^3^  Brian Tomlinson^1^  Stephen K.W. Tsui^5^  Weichuan Yu^6^  Kevin Y.L. Yip^7^  Ting Fung Chan^8^  Xiaodan Fan^9^  Wing Yee So^1,2^  Cheuk Chun Szeto^1^  Nelson L.S. Tang^3^  Andrea O. Luk^1,2,3^  Xiaoyu Tian^5^  Guozhi Jiang^1^  Claudia H.T. Tam^1^  Heung Man Lee^1^  Cadmon K.P. Lim^1^  Katie K.H. Chan^2^  Fangying Xie^1^  Alex C.W. Ng^1^  Grace P.Y. Cheung^1^  Ming-wai Yeung^1^  Shi Mai^5^  Fei Xie^1^  Sen Zhang^6^  Pu Yu^6^  Meng Weng^6^ |
| --- |
| **Affiliations** |

^1^Department of Medicine and Therapeutics, The Chinese University of Hong Kong, Hong Kong

^2^Hong Kong Institute of Diabetes and Obesity, The Chinese University of Hong Kong, Hong Kong

^3^Li Ka Shing Institute of Health Sciences, The Chinese University of Hong Kong, Hong Kong

^4^Integrated Bioinformatics Laboratory for Cancer Biology and Metabolic Diseases, The Chinese University of Hong Kong, Hong Kong

^5^School of Biomedical Sciences, The Chinese University of Hong Kong

^6^Department of Electronic and Computer Engineering, The Hong Kong University of Science and Technology

^7^ Department of Computer Science and Engineering, The Chinese University of Hong Kong

^8^ School of Life Sciences, The Chinese University of Hong Kong

^9^ Department of Statistics, The Chinese University of Hong Kong, Hong Kong
