# Supplementary material for: Obesity, clinical, and genetic predictors for glycemic progression in Chinese patients with type 2 diabetes: A cohort study using the Hong Kong Diabetes Register and Hong Kong Diabetes Biobank
Source: PLoS Med. 2020 Jul 28;17(7):e1003209. doi: 10.1371/journal.pmed.1003209 (PMC7386560; doi:10.1371/journal.pmed.1003209)
Supplement: S2 Text — (DOC) [file pmed.1003209.s019.doc]

S2 Text. Hong Kong Diabetes Biobank Study Group Members.

Ronald C.W. Ma ^1,2,3,4^

Juliana C.N. Chan ^1,2,3,4^

Risa Ozaki^1,2^

Andrea O. Luk^1,2,3,4^

Wing Yee So^1,2^

Cadmon King Poo Lim ^1,3^

Ka Fai Lee^5^

Shing Chung Siu^6^

Grace Hui ^7^

Chiu Chi Tsang ^8^

Kam Piu Lau ^9^

Jenny Y.Y. Leung ^10^

Man Wo Tsang^11^

Grace Kam^11^

Elaine Cheung^11^

Ip Tim Lau ^12^

June Kam-yin Li^13^

Vincent TF Yeung ^14^

Samuel KS Fung^15^

Stanley Lo^16^

Emmy Lau^16^

Yuk Lun Cheng^17^

Stephen Kwok-wing Tsui^18^

Yu Huang^18^

Hui-yao Lan^1,3^

Weichuan Yu^19^

Brian Tomlinson^1^

Si Lok^20^

Ting Fung Chan^21^

Kevin Yuk-lap Yip^22^

Cheuk Chun Szeto^1,3^

Xiaodan Fan^23^

Nelson LS Tang^3, 24^

Xiaoyu Tian^18^

Claudia H.T. Tam^1,4^

Guozhi Jiang^1,4^

Shi Mai^18^

Baoqi Fan^1,2,4^

Eric S Lau^1^

Fei Xie^1^

Sen Zhang^19^

Pu Yu^19^

Meng Wang^19^

Heung Man Lee^1^

Fangying Xie^1^

Alex C.W. Ng^1,4^

Grace Cheung^1^

Alice PS Kong^1,2^

Elaine Y.K. Chow^1,2^

Ming Wai Yeung^1^

Chun Chung Chow^1^

Kitty K.T. Cheung^1^

Rebecca Y.M. Wong^1^

So Hon Cheong^18^

Katie KH Chan^1,2^

Chin-san Law^12^

Anthea Ka Yuen Lock^12^

Ingrid Kwok Ying Tsang^12^

Susanna Chi Pun Chan^12^

Yin Wah Chan^12^

Cherry Chiu^2^

Chi Sang Hung^11^

Cheuk Wah Ho^11^

Ivy Hoi Yee Ng^11^

Juliana Mun Chun Fok^6^

Kai Man Lee^6^

Hoi Sze Candy Leung^14^

Ka Wah Lee^13^

Hui Ming Chan^13^

Winnie Wat^16^

Tracy Lau^16^

Rebecca Law^15^

Ryan Chan^15^

Candice Lau^1^

Pearl Tsang^1^

Vince Chan^1^

Lap Ying Ho^1^

Eva Wong^1^

Josephine Chan^1^

Sau Fung Lam^1^

Jessy Pang^1^

Yee Mui Lee^1^

^1^ Department of Medicine and Therapeutics, The Chinese University of Hong Kong

^2^ Hong Kong Institute of Diabetes and Obesity, The Chinese University of Hong Kong

^3^ Li Ka Shing Institute of Health Sciences, The Chinese University of Hong Kong

^4^ Chinese University of Hong Kong-Shanghai Jiao Tong University Joint Research Centre in Diabetes Genomics and Precision Medicine

^5^ School of Biomedical Sciences, The Chinese University of Hong Kong

^5^ Department of Electronic and Computer Engineering, Hong Kong University of Science and Technology

^6^ Department of Medicine and Geriatrics, Kwong Wah Hospital

^7^ Diabetes Centre, Tung Wah Eastern Hospital, Hong Kong

^8^ Diabetes and Education Centre, Alice Ho Miu Ling Nethersole Hospital, Hong Kong

^9^ North District Hospital, Hong Kong

^10^ Department of Integrated Medical Service, Ruttonjee Hospital, Hong Kong

^11^ Diabetes Ambulatory Care Centre, Department of Medicine and Geriatrics, United Christian Hospital

^12^ Tseung Kwan O Hospital, Hong Kong

^13^ Department of Medicine, Yan Chai Hospital, Hong Kong

^14^ Centre for Diabetes Education and Management, Our Lady of Maryknoll Hospital, Hong Kong

^15^ Department of Medicine and Geriatrics, Princess Margaret Hospital, Hong Kong

^16^ Department of Medicine, Pamela Youde Nethersole Eastern Hospital, Hong Kong.

^17^ Department of Medicine, Alice Ho Miu Ling Nethersole Hospital, Hong Kong

^18^ The Centre for Applied Genomics, The Hospital for Sick Children, Toronto, Canada

^19^ School of Life Sciences, The Chinese University of Hong Kong

^20^ Department of Computer Science and Engineering, The Chinese University of Hong Kong

^21^ Department of Statistics, The Chinese University of Hong Kong

^22^ Department of Chemical Pathology, The Chinese University of Hong Kong

^23^ CUHK-SJTU Joint Research Centre on Diabetes Genomics and Precision Medicine

^24^ Integrated Bioinformatics Laboratory for Cancer and Metabolic Diseases, The Chinese

University of Hong Kong
